# The ARE-binding protein Tristetraprolin (TTP) is a novel target and mediator of calcineurin tumor suppressing function in the skin

**DOI:** 10.1371/journal.pgen.1007366

**Published:** 2018-05-03

**Authors:** Xunwei Wu, Alice Tommasi di Vignano, Qian Zhou, Piotr J. Michel-Dziunycz, Fuxiang Bai, Jun Mi, Jing Qin, Tingjian Zu, Günther F. L. Hofbauer

**Affiliations:** 1 Laboratory for Tissue Engineering and Regeneration and Shandong Provincial Key Laboratory of Oral Tissue Regeneration, School of Stomatology, Shandong University, Jinan, Shandong, China; 2 Cutaneous Biology Research Centre, Massachusetts General Hospital, Charlestown, MA, United States of America; 3 Department of Dermatology, University Hospital Zurich, Zürich, Switzerland; Brigham and Women's Hospital, UNITED STATES

## Abstract

An increased incidence of skin inflammatory diseases is frequently observed in organtransplanted patients being treated with calcineurin inhibitor-based immunosuppressive agents. The mechanism of increased skin inflammation in this context has however not yet been clarified. Here we report an increased inflammation following inhibition of calcineurin signaling seen in both chemically induced mouse skin tumors and in tumors grafted from *H-ras*^*V12*^ expressing primary human keratinocytes (HKCs). Following UVB or TPA treatment, we specifically found that deletion of the calcineurin gene in mouse keratinocytes (MKCs) resulted in increased inflammation, and this was accompanied by the enhanced production of pro-inflammatory cytokines, such as TNFα, IL-8 and CXCL1. Furthermore, expression of the RNA-binding protein, tristetraprolin (TTP) was down-regulated in response to calcineurin inhibition, wherein TTP was shown to negatively regulate the production of pro-inflammatory cytokines in keratinocytes. The induction of TTP following TPA or UVB treatment was attenuated by calcineurin inhibition in keratinocytes, and correspondingly, disruption of calcineurin signaling down-regulated the amounts of TTP in both clinical and *H-ras*^*V12*^-transformed keratinocyte tumor models. Our results further demonstrated that calcineurin positively controls the stabilization of TTP in keratinocytes through a proteasome-dependent mechanism. Reducing the expression of TTP functionally promoted tumor growth of *H-ras*^*V12*^ expressing HKCs, while stabilizing TTP expression counteracted the tumor-promoting effects of calcineurin inhibition. Collectively these results suggest that calcineurin signaling, acting through TTP protein level stabilization, suppresses keratinocyte tumors by downregulating skin inflammation.

## Author summary

It is known that a higher incidence of skin squamous cell carcinoma (SCC) development, as well as increased chronic skin inflammation, occurs in organ transplant patients with long-term use of calcineurin inhibitors as immunosuppressive drugs. Whether the increased skin inflammation contributes to the development of SCC in these patients is not clear, and importantly, the mechanism of increased skin inflammation with long-term administration of immunosuppressive drugs has not yet been clarified. Here we report that the disruption of calcineurin signaling results in an increased inflammatory response in the skin. The increased skin inflammation was associated with the down-regulation of a RNA-binding protein, Tristetraprolin (TTP), also known as zinc finger protein 36 homolog (ZFP36). We further demonstrated that calcineurin controls the stabilization of TTP in the skin and the expression of TTP promotes keratinocyte tumorigenesis, while high expression levels of TTP counteract the pro-tumorigenic effects of calcineurin inhibition. Our study is the first to discover that TTP is a novel target of calcineurin and plays a tumor-suppressing role in the skin. Further, our data suggest that inflammation may be an important factor in the development of SCC in organ transplant patients.

## Introduction

Calcineurin is the only known serine/threonine phosphatase under calcium/calmodulin control [1]. The active enzyme is a heterotrimeric complex formed by a larger catalytic subunit (calcineurin A, CnA), a Ca^2+^-binding regulatory subunit (calcineurin B, CnB) and calmodulin [2]. CnB is expressed in two isoforms, CnB1 (ubiquitous) and CnB2 (testis-specific). Therefore, the inhibition of CnB1 in the skin could completely block calcineurin signaling since only CnB1 is expressed in keratinocytes [3]. The activation of calcineurin dephosphorylates and thus activates Nuclear Factors of Activated T cells (NFATs), which controls the expression of multiple genes related to development and growth, immune responses and inflammatory responses [4, 5]. The function of calcineurin in the immune system has been elucidated in great detail, where it plays a key role in regulating T and B cell development, and is an important pharmacological target for immune suppression [6]. Cyclosporin A (CsA), which binds and suppresses calcineurin activity in a complex with the cellular protein cyclophilin A [7, 8], has been mainly used as an anti-rejection drug for organ transplanted patients [9]. It is known that the higher incidence of skin squamous cell carcinoma (SCC) development, as well as increased chronic skin inflammation, occurs in organ transplant patients with long-term use of CsA [10–12]. Several mechanisms have been shown to contribute to the development of SCC in these patients, and increased levels of smoldering inflammation may also be important factors [10, 11]. However, the mechanism underlying increased skin inflammation with long-term administration of immunosuppressive drugs has not yet been clarified.

Inflammation has been shown to play an important role in different stages of tumor development, including initiation, promotion and metastasis [13, 14]. Inflammation is mainly mediated by the production of cytokines, which recruit immune cells to trigger the inflammatory response. Epidermal keratinocytes, due to their strategic position at the interface between the body and the environment, are the most important type of cell that responds to external stimuli, and usually initiates the inflammatory response by regulating cytokine production during the development of skin inflammation. Cytokine production is controlled at various stages of gene expression, including transcription, mRNA export, and post-transcriptional and translational levels. Recent studies have suggested that the post-transcriptional regulation of RNA stability plays a vital role in controlling the expression of cytokines [15]. The stability of mRNA is mediated mainly by proteins that bind to AU-rich elements (AREs) located in 3’-untranslated regions (3’UTRs) of mRNA. A number of ARE-binding proteins have been identified that regulate the stability of mRNA, including tristetraprolin (TTP), human antigen-related protein (HuR, T-cell-restricted intracellular antigen-1 (TIA-1), and TIA-related protein (TIAR) [15, 16]. Among those, the zinc finger protein, TTP (ZFP36) encoded by the ZFP36 gene, which belongs to the TIS11/TTP gene family, is the best-characterized [16].

TTP has been reported to regulate the stability of multiple target mRNAs, including pro-inflammatory cytokines and chemokines, such as TNFα, IL-6/IL-8, GM-CSF and CXCL1 [16–18]. TTP-deficient mice exhibit a severe inflammation syndrome characterized by growth retardation, cachexia, polyarthritis, autoimmunity, myeloid hyperplasia and dermatitis [19]. TTP is believed to promote the ARE-mediated decay of cytokine mRNA [16, 20]. Recent studies have suggested that TTP plays a tumor suppressing function in cancer development [18, 21–23]. The deregulation of TTP has been found to play an important role in the progression of various cancers, including inflammation-related cancers, as well as in processes of proliferation, apoptosis, angiogenesis, metastasis, invasion and chemotherapy resistance [18, 22]. Heretofore, the physiological role of TTP has been mainly assessed in myeloid cells, such as macrophages or dendritic cells (DCs). Recently TTP expression was found in keratinocytes, and a TTP deficiency in keratinocytes resulted in the increased expression of transcripts encoding pro-inflammatory cytokines and chemokines in the skin. The sum of these data suggests that TTP plays a crucial role in the regulation of cytokine production by keratinocytes [24]. However, it still not clear how TTP is activated and what its functions in keratinocytes might be.

Emerging data suggest that calcineurin signaling plays a tumor suppressing function in keratinocytes [25]. Our previous study showed that the disruption of calcineurin/NFAT signaling promotes keratinocyte tumorigenesis by blocking p53-dependent cellular senescence, and the accompanying histological findings revealed that there is an increased inflammation response surrounding tumors when calcineurin signaling is inhibited [26]. The goal of the present study was to test whether calcineurin controls keratinocyte tumorigenesis through the regulation of skin inflammation and to explore its underlying mechanism. We now report that calcineurin negatively controls cytokine production by stabilizing TTP, a novel target of calcineurin, and thereby suppresses keratinocyte tumor formation.

## Results

### Loss of calcineurin signaling increases the inflammatory response in skin tumors

We previously showed that disruption of calcineurin signaling, such as by deletion of the *CnB1* gene, or treatment with the calcineurin inhibitor CsA, promotes keratinocyte tumorigenesis by blocking p53-mediated cellular senescence [26]. We also noted an increased inflammatory response surrounding these tumors. To confirm that observation, immunofluorescence (IF) staining of inflammatory cells using the Gr-1 (Ly6) antibody for granulocytes (neutrophils) and the F4/80 antibody for macrophages, showed significantly increased infiltration of inflammatory cells into tumors formed in mice with or without the CnB1 deletion (KO) (S1A and S1B Fig). For analysis of the possible infiltration of adaptive immune cells in the tumors, we performed IF staining of different types of T cells with antibodies to CD4 and to CD8 for T cells, to CD16 for NK cells and to CD161 for Treg cells (S2A–S2D Fig). We observed the presence of T cells both in wild-type control (Ctrl) and in CnB1 KO (KO) tumors, and there was no significant difference between them, indicating that adaptive immune cells didn’t play a major role in the increased inflammation of CnB1 KO skin tumors. Increased inflammation was also identified by analysis of tumor xenografts, derived from our previous study [26], formed by *H-ras*^*V12*^ expressing primary human keratinocytes (HKCs) with concomitant CnB1 knockdown (siCnB1) or in mice subsequently treated with CsA (S3A and S3B Fig). These data suggested that the disruption of calcineurin signaling could result in the up-regulation of innate cells-mediated inflammation in skin tumors.

### The disruption of cacineurin signaling in skin keratinocytes enhances the acute inflammatory response induced by TPA or UVB

**Fig 1:**
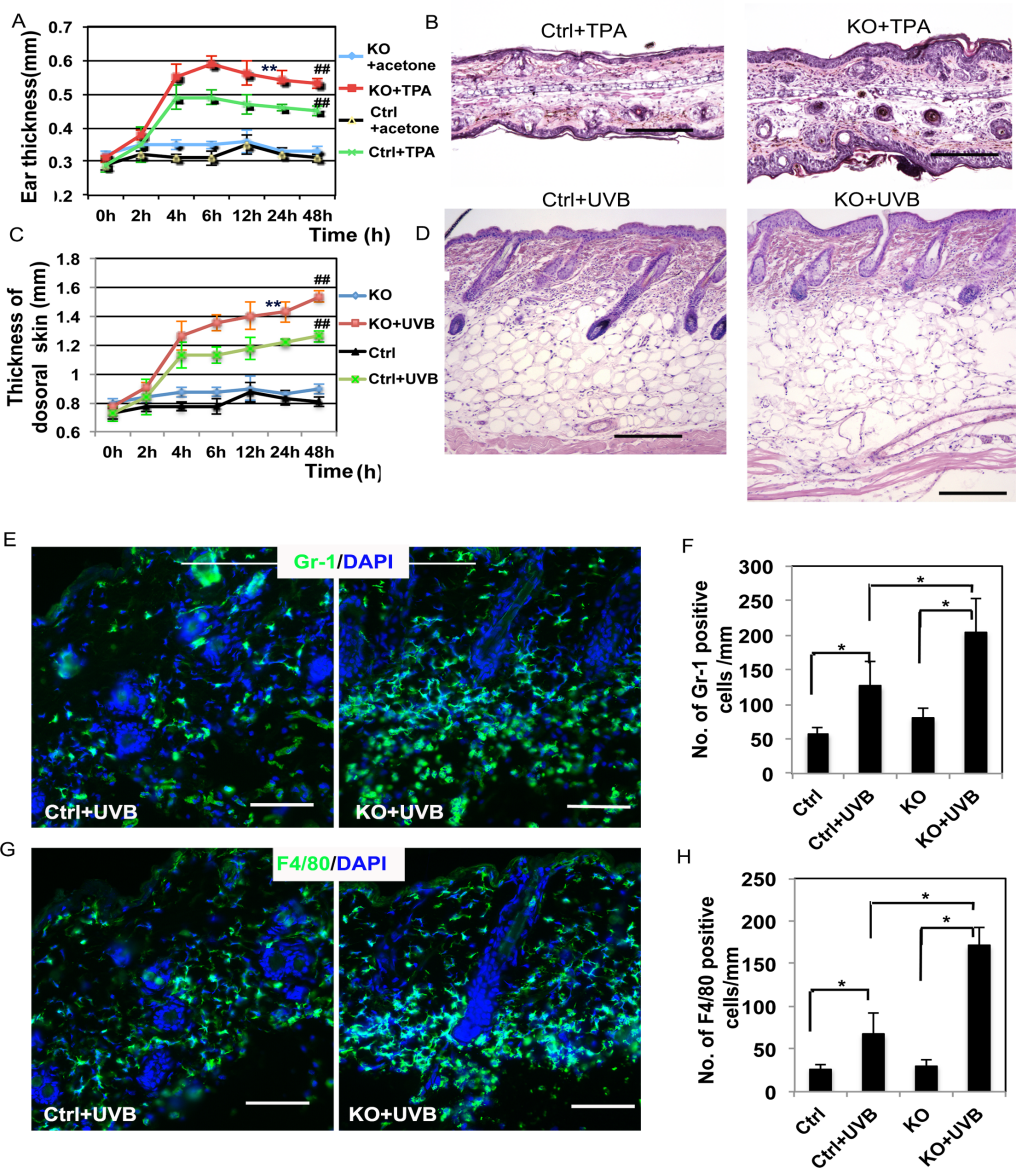
Deletion of CnB1 in keratinocytes enhances the skin acute inflammatory response to TPA treatment and to UVB exposure. **A**. Ear thickness was measured at different time points as indicated. ## p<0.01 when comparing the TPA-treated group with the untreated group, ** p<0.01 when comparing the CnB1-/- (KO) group (KO+TPA) with the control CnB1+/+ group (Ctrl+TPA); **B.** Histological analysis of ears of Ctrl and KO mice at 48 hr after TPA treatment; **C.** Thickness of dorsal skin collected at the indicated time points was measured using a microscope. ## p<0.01 when comparing the UVB-exposed group with the untreated group, ** p<0.01 when comparing the KO group (KO+UVB) with the control group (Ctrl+UVB); **D.** Histological analysis of dorsal skins of Ctrl and KO mice at 48 hr after exposure to UVB; **E-H.** Sections from **C** and **D** were analyzed for infiltration of inflammatory cells by IF analysis of Gr-1 (**E,** green) and F4/80 (**G**, green), DAPI is used as a counter-stain for nuclei. The corresponding quantification analysis of **E** and **G** is shown in **F** (Gr-1) and **H** (F4/80). * p<0.05 when comparing the data indicated with the brackets. Bars = 500 μm for **B, D**; 100 μm for **E, G**. Student’s t test analysis was used for all quantification data to compare two groups as indicated, n = 3, Standard error bars are provided in **A, C, F** and **H**.

In order to verify that the increased inflammatory response in the surrounding tissue of the generated tumors was directly due to the disruption of calcineurin signaling, TPA or UVB radiation were used to induce acute skin inflammation both in wild-type mice (Ctrl) and in mice with keratinocyte-specific deletion of the *CnB1* gene (KO). Firstly, the pro-inflammatory compound TPA was applied on the ears of mice, after which the thickness of each ear was measured at different time points (Fig 1A). Ear thickness increased significantly after TPA treatment in KO mice (KO+TPA) compared to the control group (Ctrl+TPA) (Fig 1A). Histological analysis (HE stain) showed that TPA treatment increased ear thickness with an enhanced infiltration of inflammatory cells (Fig 1B). Similar results were obtained when mouse dorsal skin was exposed to UVB and the skin was collected at different time points to analyze its thickness (Fig 1C) and its histology (Fig 1D). We also found an increased thickness of subcutaneous edema and a more severe inflammation in CnB1 KO skin compared to the control group (Fig 1C and 1D). To further confirm the increased infiltration of inflammatory cells in the skin of mice with a CnB1 deletion, IF analysis showed significantly more Gr-1 or F4/80 positive cells in CnB1KO skin after UVB exposure for 48 hr compared to the wild-type (Ctrl) controls (Fig 1E–1H). No increase of adaptive immune response was observed in Ctrl or KO dorsal skin treated with TPA for 48 hr by IF analysis of different T cell populations (S4A–S4D Fig). These data suggest that calcineurin signaling negatively controls skin inflammation, which is mainly mediated by innate immune cells in keratinocytes.

### The enhanced inflammatory response induced by calcineurin signaling disruption is accompanied by increased cytokine production by keratinocytes

**Fig 2:**
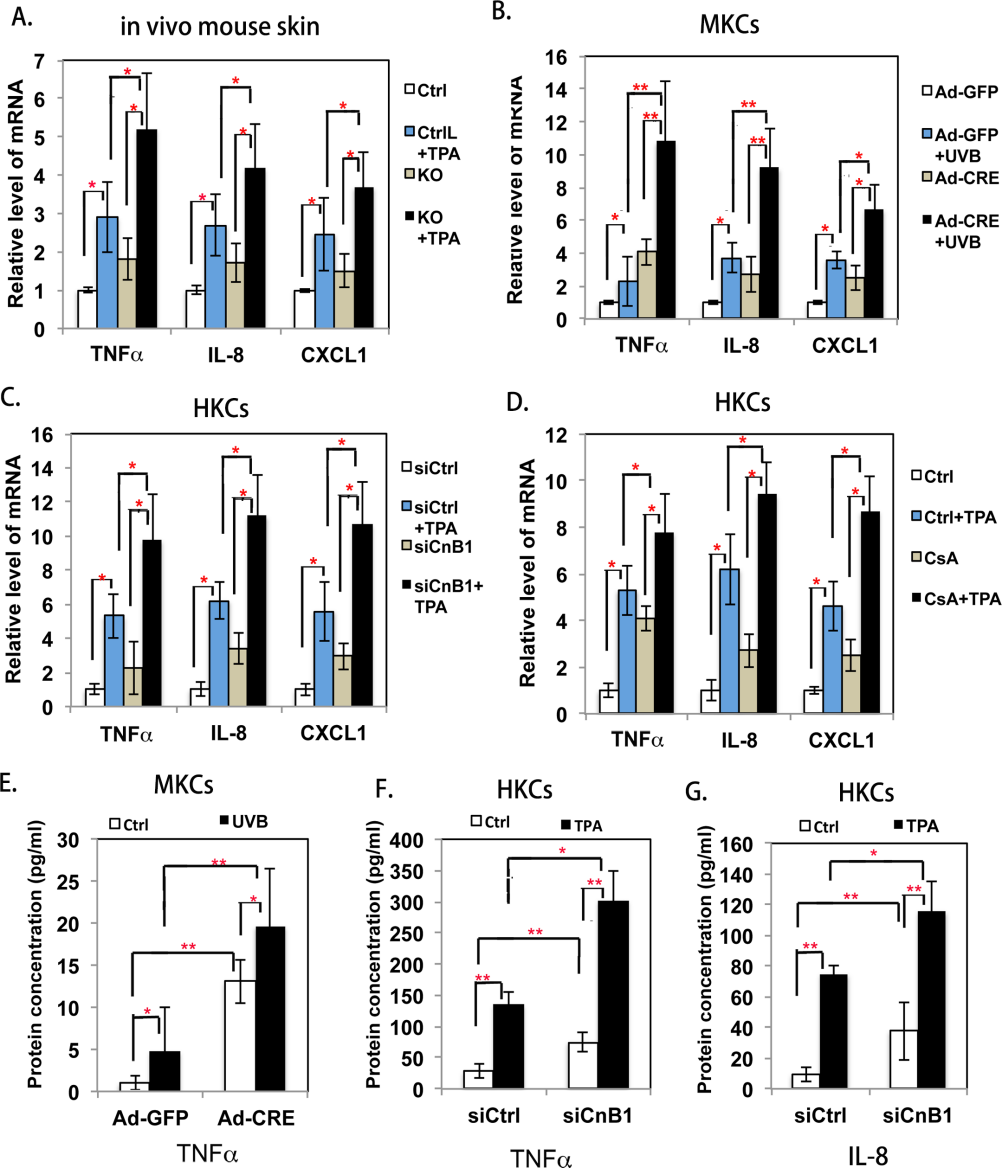
Inhibition of calcineurin signaling induces the expression of pro-inflammatory cytokines in keratinocytes. **A-D**: qRT-PCR analysis of TNFα, IL-8 and CXCL1 expression; results were normalized to levels of 36B4 mRNA. **A.** Total mRNA isolated from mouse ears from the experiments shown in Fig 1A; **B.** Total mRNA extracted from primary MKCs from CnB1f^flox/flox^ mice infected with either Ad-Cre or Ad-GFP viruses, with or without UVB treatment; **C.** Total mRNA isolated from TPA-treated HKCs transfected with siRNA of CnB1 (siCnB1) or a control scramble siRNA (siCtrl) at 24 hr; **D.** Total mRNA from HKCs treated with CsA or DSMO (Ctrl) for 24 hr, followed by treatment with TPA for another 24 hr. **E-G:** ELISA analysis of indicated cytokine protein levels in the medium. **E.** Conditioned medium collected from MKC cultures shown in **B**; **F,G**. Conditioned medium collected from HKC cultures shown in **C**. Student’s t-test was used for statistical analysis in **A-G**, standard error bars provided, * p<0.05, **p<0.01, n = 3.

Since inflammatory cell infiltration is mediated by cytokines, we next analyzed whether the increased skin inflammation found after disrupting calcineurin signaling was associated with an increased production of cytokines by keratinocytes. First, we collected ear skin treated with TPA, and analyzed the expression levels of mRNAs encoding pro-inflammatory cytokines, such as TNFα, IL-8 and CXCL1, using qRT-PCR analysis. Fig 2A shows that treatment with TPA increased the mRNA levels of all 3 pro-inflammatory cytokines, and much higher mRNA levels of cytokines were seen in CnB1 KO skin, which correlated exactly with the increased inflammatory response seen in Fig 1A and 1B. Secondly, we isolated and cultured primary CnB1^flox/flox^ mouse keratinocytes (MKCs) and then infected them with a Cre expressing adenovirus (Ad-Cre) to obtain CnB1 KO cells. The control cells were infected with a GFP expressing virus (Ad-GFP), then both types of cells were exposed to UVB for 48 hr and analyzed by qRT-PCR for pro-inflammatory cytokines. Fig 2B shows that Cre-mediated deletion of the *CnB1* gene enhanced TNFα, IL-8 and CXCL1 expression induced by UVB. Similar results were found using HKCs after the disruption of calcineurin signaling either by siRNA mediated knockdown of CnB1 (siCnB1) (Fig 2C) or by CsA to block calcineurin activity (Fig 2D). To further confirm that the inhibition of calcineurin signaling increases the production of cytokines, we performed ELISA assays to measure the protein concentrations of secreted cytokines. We found an increased TNFα protein level in MKCs after the deletion of CnB1 (Fig 2E) under both basal conditions and after UVB treatment. Levels of TNFα and IL-8 were significantly increased in HKCs after the knockdown of CnB1 under basal conditions and after TPA treatment (Fig 2F and 2G). Taken together, these data suggested that the inhibition of calcineurin signaling could enhance the production of pro-inflammatory cytokines in keratinocytes *in vivo* and *in vitro*.

### Inhibition of keratinocyte calcineurin *in vivo* and *in vitro* results in the down-regulation of TTP

**Fig 3:**
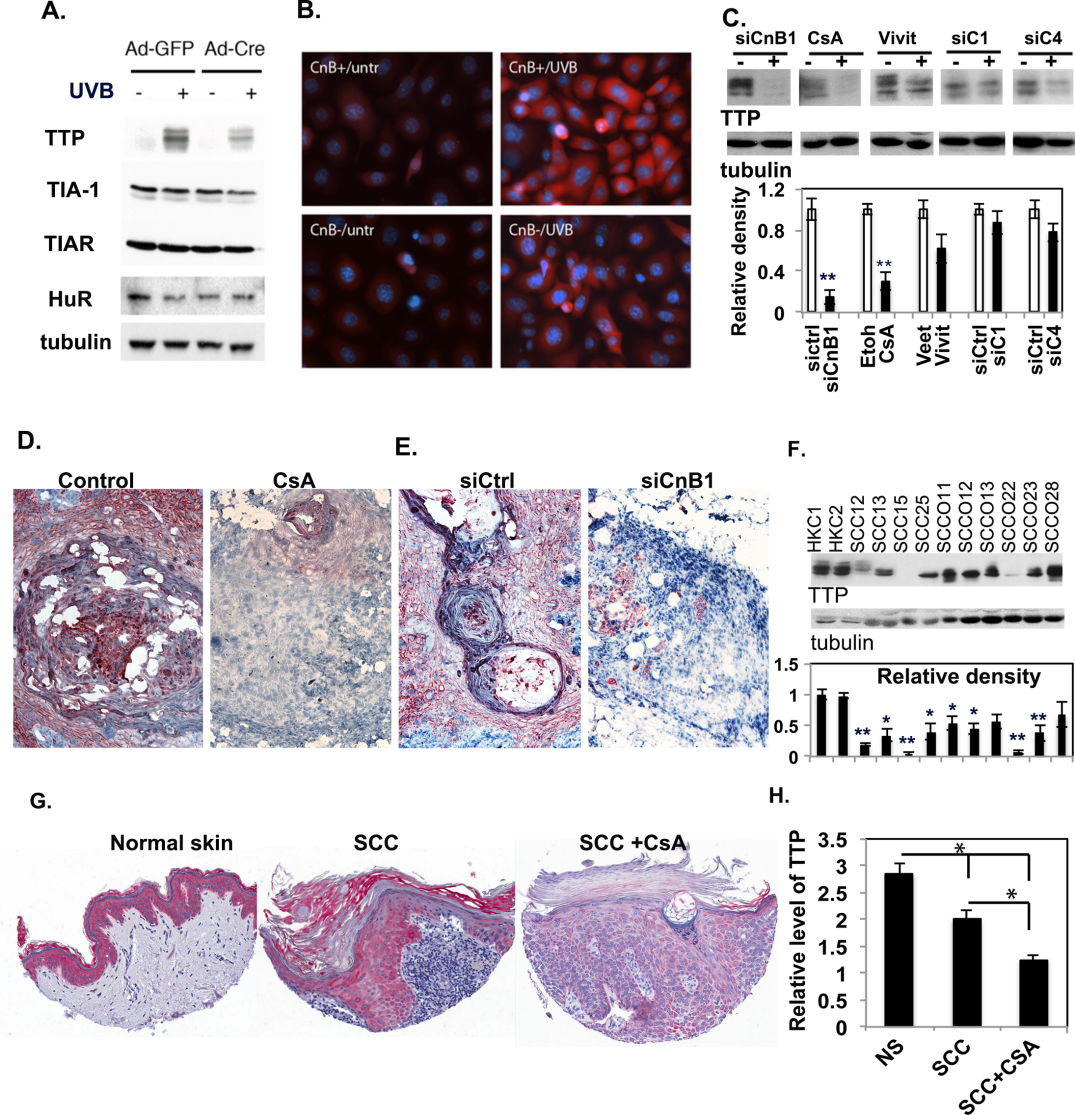
TTP is down-modulated in keratinocytes and in tumor tissues when calcineurin signaling is inhibited. **A.** Ad-GFP- or Ad-Cre-infected CnB1^flox/flox^ MKCs were irradiated with UVB (+) or were sham-irradiated (-). Four hr after irradiation, the cells were analyzed by immunoblot for TTP, TIA-1, TIAR and HuR proteins, and tubulin as a loading control. **B.** Ad-GFP (*CnB+*) and Ad-Cre infected (*CnB-*) CnB1^flox/flox^ MKCs were sham-irradiated (*untr*) or were UVB-treated (*UVB*); after 4 hr they were stained with an anti-TTP antibody (red) and DAPI (blue) for nuclei. **C.** HKCs were either transfected with siRNA of CnB1 (siCnB1), NFATC1 (siC1), siNFATc4 (siC4) or a control scramble siRNA, or were treated with Vivit (Veet as control) or with CsA (DMSO as a control). After 72 hr, cells were lysed and immunoblotted for TTP, with tubulin as a loading control. The quantification of band density is shown in the lower panel. ** indicates p<0.001. **D,E.** IHC analysis of TTP in tumor sections from S2A Fig; **F**. Immunoblot analysis of TTP from 10 different SCC lines and two primary HKC lines; tubulin as a loading control. The full-blot image is shown in S5 Fig. Quantification of band densities is shown in the lower panel. *: p<0.05, **p<0.01 when compared to primary HKCs. n = 3. **G-H.** Tissue microarrays (TMAs) of cutaneous SCCs from patients with or without CsA treatment versus the normal skin (NS) from general untreated population were analysed for TTP expression by immunohistochemistry. The quantification of TTP is shown in **H**, * indicates p<0.05 when compared with each other as indicated.

The regulation of cytokines can occur at various levels, and recent studies have suggested that post-transcriptional regulation plays a vital role in controlling their production by modulating mRNA stability through RNA binding proteins. We hypothesized that the negative regulation of cytokine expression by calcineurin signaling in skin keratinocytes is probably through post-transcriptional regulation. To test that hypothesis, primary CnB1^flox/flox^ MKCs infected with Ad-GFP or Ad-Cre viruses and irradiated with UVB were analyzed by Western-blot to determine levels of RNA binding proteins that have been shown to bind ARE and control the stability of 4 cytokine mRNAs: TTP, TIA-1, TIAR and HuR. We found that, although UVB strongly induces TTP expression in both genotypes, CnB1-deficient keratinocytes express a significantly lower amount of TTP, but the 3 other ARE-binding proteins TIA-1 TIAR and HuR were not affected (Fig 3A and S5A Fig). The down-regulation of TTP in CnB1-deficient primary keratinocytes was also confirmed by immunofluorescence staining with a TTP antibody (Fig 3B and S5B Fig).

To further test whether TTP down-regulation in HKCs is caused by the disruption of calcineurin signaling, we performed western-blot analysis of TTP in HKCs with blocking of calcineurin by siRNA of CnB1 (siCnB1) or with CsA or by inhibiting the downstream target of calcineurin by knockdown of NFATc1 (siC1) or NFATc4 (siC4) or by the NFAT inhibitor VIVIT. As shown in Fig 3C, the direct inhibition of calcineurin with a siRNA of CnB1 or with CsA significantly down-regulated the TTP level. In contrast, blocking the calcineurin downstream target NFATs also reduced the level of TTP, but not at a statistically significant level, suggesting that the regulation of TTP level in keratinocytes is likely through calcineurin B activity. Consistent with that observation in vitro, we found that TTP expression was reduced in tumors formed by injection of ras-expressing HKCs in mice treated with CsA (Fig 3D) and in tumors formed by ras-expressing HKCs with knockdown of CnB1 by siRNA (siCnB1, Fig 3E). We also found a lower expression of TTP in most skin and oral SCC cell lines compared with normal primary keratinocytes *in vitro* (Fig 3F, S6 Fig). Importantly, lower TTP levels were found in tissue microarrays (TMAs) of clinically excised SCC tumors from immunocompetent patients undergoing CsA treatment compared to those without CsA treatment, and also compared to normal skin (NS) derived from immunocompetent patients without SCCs (Fig 3G and 3H). In summary, these data suggest that the disruption of calcineurin in keratinocytes or in SCC cells, *in vitro* and *in vivo*, down-regulates the expression of TTP.

### TTP negatively controls the production of pro-inflammatory cytokines by keratinocytes

**Fig 4:**
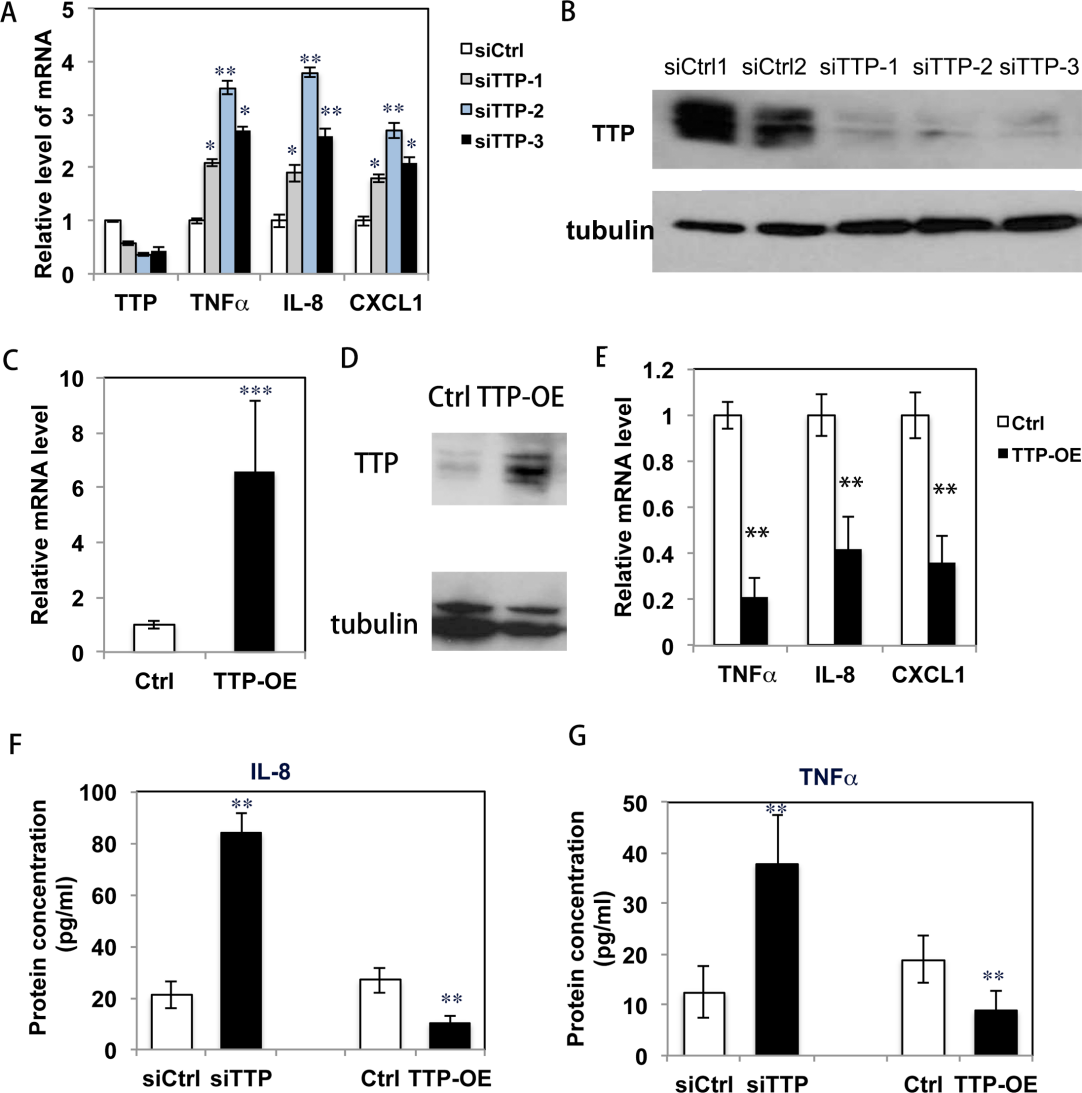
TTP negatively regulates cytokine production in keratinocytes. **A.** HKCs were transfected with 3 individual siRNAs of TTP (siTTP-1, -2, -3), and 72 hr after transfection, cells were analyzed for TTP and cytokines (TNFα, IL-8, CXCL1) expression by qRT-PCR; expression levels were normalized against the 36B4 gene. * p<0.05, ** p<0.01 when comparing the TTP knockdown group with the control group (siCtrl). **B.** Cells analyzed by immunoblot for TTP protein level with tubulin as a loading control. The image of full-blot of **B** is shown in S7 Fig; **C**. HKCs were infected with a TTP expressing lentivirus (TTP-OE); 72 hr after infection, cells were analyzed for TTP mRNA by qRT-PCR. **D.** TTP protein levels analyzed by immune-blotting. **E**. Cytokine expression analyzed by qRT-PCR. ** indicates p<0.01 when comparing cells in the TTP-OE group to the control group (Ctrl). **F,G**. Conditioned media were collected from the TTP over-expressing cells cultured for 72 hr for analysis of IL-8 **(F)** and TNFα **(G)** level by ELISA. *: p<0.05 and **:p<0.01. All experiments were repeated 3 times.

Next, we tested whether TTP controls cytokine production by keratinocytes. First, when TTP expression was knocked down by siRNA in HKCs, mRNA levels of TNFα, IL-8 and CXCL1 were up-regulated (Fig 4A). The efficiency of TTP knockdown was confirmed by western-blot analysis (Fig 4B and S7 Fig). The results recapitulated what was found in keratinocytes after calcineurin was inhibited. Second, when HKCs were infected with a lentivirus expressing a full length TTP cDNA devoid of the 3’ UTR region to over-express TTP (TTP-OE) (Fig 4C and 4D), the high expression of TTP caused a decreased production of pro-inflammatory cytokines (Fig 4E). We confirmed that protein levels of cytokines (IL-8 and TNFα) were induced in keratinocytes following the knockdown of TTP and were reduced in keratinocytes that over-expressed TTP using ELISA analysis (Fig 4F and 4G).

### CnB1 regulates TTP stabilization in keratinocytes through a proteasome degradation mechanism

**Fig 5:**
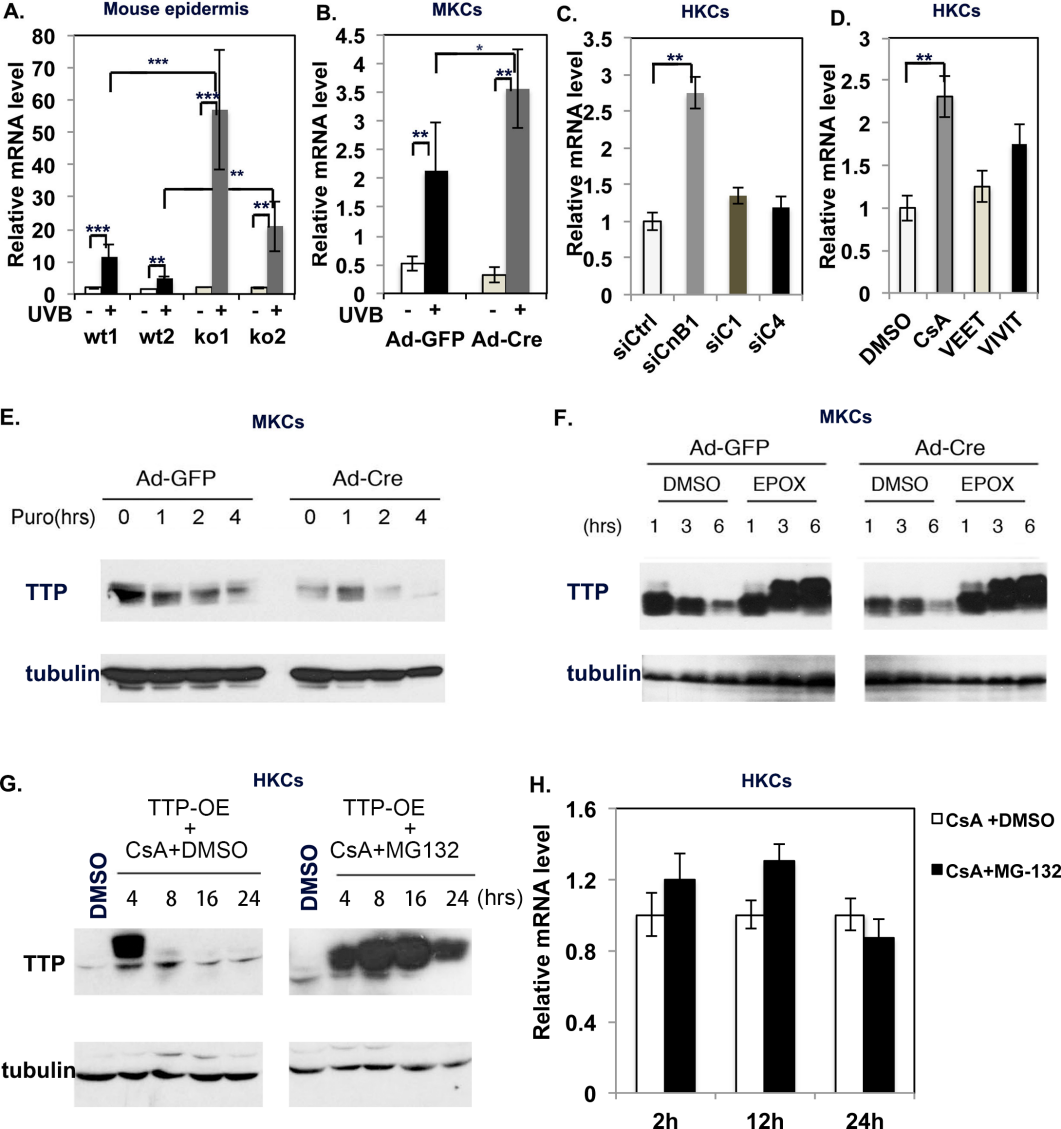
CnB1 regulates TTP stability through proteasomal mediation. **A.** qRT-PCR analysis of TTP expression level from the dorsal epidermis of two wild-type (*wt*) and two CnB1-/- (*KO*) mice 24 hr after treatment with UVB (+) or sham-irradiation (-) as control. **B.** Ad-GFP- or Ad-Cre-infected CnB1^flox/flox^ MKCs were treated with UVB (+) or sham-irradiated (-). Four hours after treatment, cells were analyzed for TTP mRNA level by qRT-PCR. **C,D.** HKCs were transfected with siRNAs of scramble RNA (siCtrl), calcineurin B1(siCnB1), NFATc1(siC1) or NFATc4 (siC4) or were treated with DMSO, CsA, Veet or Vivit. 48 hr after treatment, cells were analyzed for TTP mRNA level by qRT-PCR. **A-D**: Normalized to 36B4 gene, *p<0.05, **p<0.01, ***p<0.001; n = 3. **E.** Ad-GFP- or Ad-Cre-infected CnB1^flox/flox^ MKCs, UVB-irradiated as in **B,** were incubated with puromycin (*PURO*) or DMSO (*ctrl*). Cells were then collected at the indicated times and analyzed by immuoblot for TTP (tubulin as loading control). **F.** Ad-GFP- or Ad-Cre-infected CnB1-loxP MKCs were treated with UVB as in **B,** in the presence of epoxomicin 1μM (*EPOX*) or *DMSO* at the indicated times. Cells were then analyzed by immunoblot for TTP, tubulin as a loading control. **G.** HKCs with over-expression of TTP (TTP-OE) with treated either with CsA with or without MG132 were collected at indicated times for analysis of TTP protein level. **H.** qRT-PCR analysis of TTP mRNA level at indicated time points in HKCs treated with CsA with or without MG132.

In order to investigate the underlying mechanism by which calcineurin inhibition down-regulates TTP, we first analyzed the levels of TTP mRNA using qRT-PCR analysis. We found that TTP mRNA levels actually increased in MKCs with the deletion of CnB1 (Fig 5A and 5B), and this result was confirmed in HKCs by knockdown of CnB1 (siCnB1) and by blocking CnB1 activity with CsA (Fig 5C and 5D). However, the inhibition of NFATs either by Vivit or by knockdown of NFATc1 or NFATc4 didn’t significantly affect the mRNA level of TTP. These data suggested that calcineurin likely regulates the stabilization of TTP protein. In order to test that possibility, we sequentially treated MKCs with UVB to induce TTP expression and with puromycin to prevent novel protein synthesis. We found that in CnB1-deleted cells, TTP protein was significantly destabilized (Fig 5E), and its half-life was clearly reduced (94 min; controls: 147 min) (S8 Fig).

To assess the involvement of proteasome-mediated degradation in that process, we analyzed TTP expression in the presence of the potent proteasome inhibitor Epoxomicin (Fig 5F). The differential expression of TTP observed in the control versus KO cells was completely abolished by treatment with Epoxomicin, which suggests that calcineurin controls TTP stability through a mechanism involving proteasomes (Fig 5F). In further support of this mode of regulation, we infected HKCs with a TTP over-expressing lentivirus (TTP-OE, see Fig 4D), after which the stably infected keratinocytes were treated with CsA or with DMSO vehicle alone, followed by immunoblotting to determine TTP protein levels. As shown in Fig 5G, even in TTP over-expressing keratinocytes, CsA treatment caused a strong down modulation of TTP, which was counteracted by the proteasome inhibitor MG132. Finally, qRT-PCR analysis showed no significant difference in the TTP mRNA level between CsA treated or CsA plus MG132-treated HKCs (Fig 5H). Taken together, these data suggested that calcineurin regulates the stabilization of TTP protein likely through the proteasome degradation pathway.

### TTP, as a downstream target of CnB1, plays a tumor-suppressing role in keratinocytes *in vivo*

**Fig 6:**
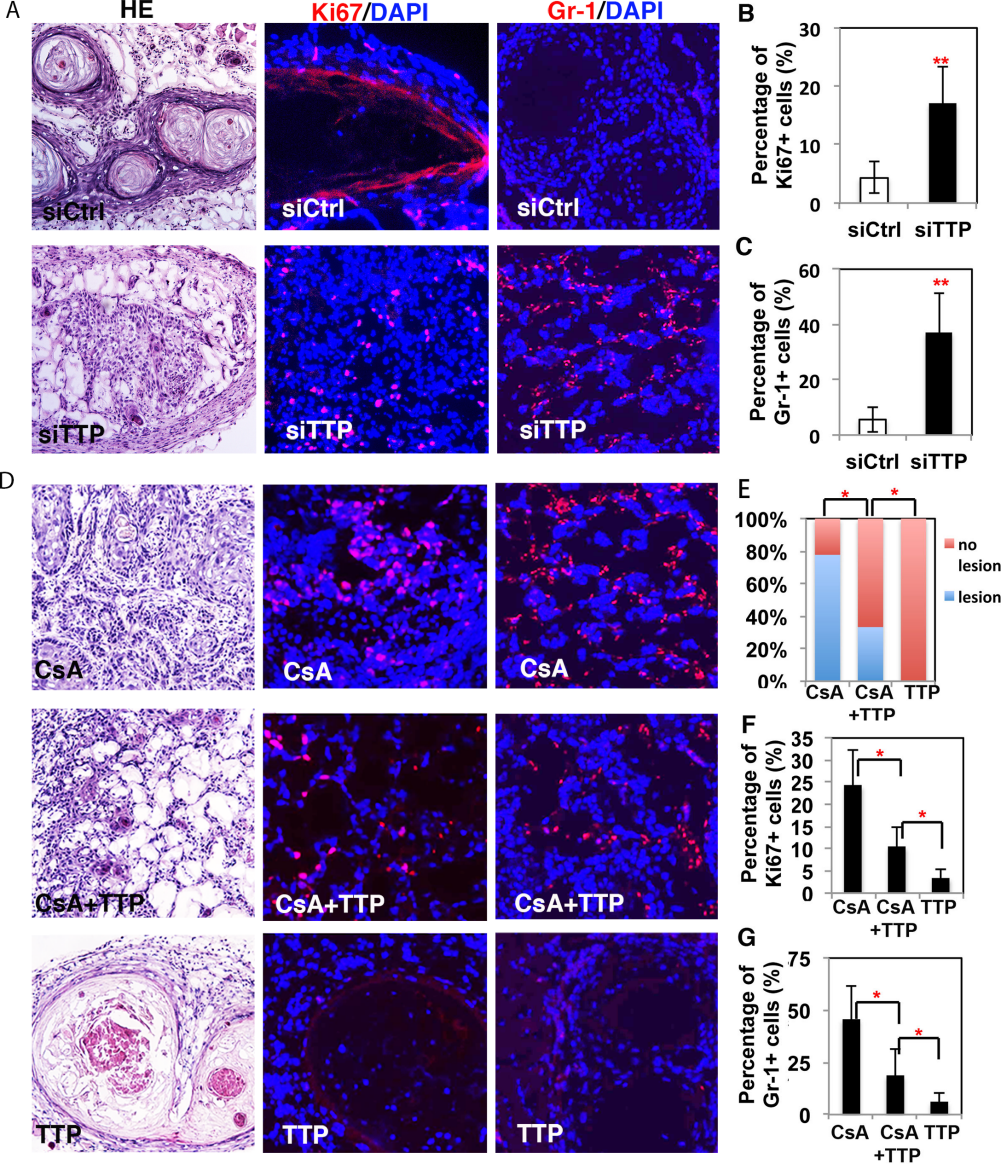
TTP plays a tumor-suppressing function in keratinocytes. **A.** H-*ras*^V12^ expressing HKCs with siRNA-mediated TTP knockdown (siTTP) versus control (siCtrl) were injected into the dermal-epidermal junction of nu/nu mouse skin. Mice were sacrificed 6 wks later for histological analysis and IF staining of Ki67 and Gr-1. Results were quantified by establishing the percentage of Ki67 (**B**) and Gr-1 (**C**) positive cells over DAPI positive cells. **D.** Similar assays were performed with H-*ras*^V12^ expressing HKCs with or without stable expression of TTP, followed by treatment with CsA or DMSO. Four wks later, the mice were sacrificed for histological analysis and Ki67 (**F**) and Gr-1 (**G**) positive cells were counted. According to the histological analysis, the quantification of lesions was shown in **E**. The results were quantified by calculating the percentage of Ki67 (**F**) and Gr-1 (**G**) positive cells divided by DAPI positive cells. **B, D, F, G**: *p<0.05, ** p<0.01, n = 3.

To assess the biological significance of the down-modulation of TTP expression on CsA-tumor promoting effects, a dual approach was undertaken. The siRNA-mediated knockdown of TTP expression was found to enhance the tumorigenicity of *H-ras*^*V12*^ expressing HKCs, tested by intradermal injection into nu/nu mice. Grafts with injected *H-ras*^*V12*^ expressing HKCs with siTTP showed higher cellularity, an increased Ki67 labeling index and increased surrounding cellular inflammation compared to the controls (Fig 6A–6C). Conversely, *H-ras*^*V12*^ expressing HKCs infected with a TTP over-expressing lentivirus and injected intradermally into nu/nu mice followed by CsA treatment exhibited a significantly lower tumorigenic phenotype (decreased cellularity, decreased Ki67 labeling index and decreased surrounding cellular inflammation) than *H-ras*^*V12*^ expressing HKCs infected with an empty vector control virus (Fig 6D–6F). HKCs that over-expressed TTP did not give rise to lesions (Fig 6G). These results suggest that the over-expression of TTP at least partially counteracts the tumor formation induced by *ras*^*V12*^ expressing HKCs treated with CsA. Taken together, these data indicate that TTP is a downstream target of calcineurin signaling and plays a tumor-suppressing role in keratinocytes.

Next, we sought to further understand the mechanism of the tumor-suppressing function of TTP in the skin since increased inflammation was observed in grafts with knockdown of TTP in keratinocytes. We analyzed changes of cytokine expression and of metabolic genes, which have been reported to play crucial roles in TTP tumor-suppression function[18, 23], in SCC13 skin tumor cells with modulations of TTP expression levels. We found that the knockdown of TTP in SCC13 cells induced the expression of the cytokines IL-8, TNFα, VEGF and COX2 (S9A Fig) as well as genes involved in metabolic pathways including the pyruvate dehydrogenase complex (PDK1), the citric acid cycle (IDH3A), the electron transport chain (GPD2), branched-chain amino acid metabolism (BCKDHB), purine biosynthesis (ADSS) and the pyrimidine salvage pathway (CMPK1) (S9B Fig). In contrast, the over-expression of TTP reduced the expression of cytokines and metabolic genes in SCC13 cells (S9C and S9D Fig).

## Discussions

### Calcineurin signaling controls skin inflammation by negatively regulating cytokine production in keratinocytes

First we observed increased inflammation surrounding the experimental mouse and human skin tumors derived in our previous study [26] that were induced by the disruption of calcineurin signaling. We further found that the pro-inflammation stimuli of TPA treatment and of UVB exposure induced more acute skin inflammation in mice with the *CnB1* gene deleted in keratinocytes. Calcineurin, through its downstream transcriptional factor NFATs, plays a crucial role in regulating T cell and B cell development [1, 6]. However, our results show that the increased inflammation is mainly mediated by innate immune cells, not by adaptive immune cells in skin with deletion of the *CnB1* gene in keratinocytes. The skin inflammation is initiated and mediated by keratinocytes due to their strategic position at the outermost layer of the skin, and keratinocytes communicate with other cells by means of cytokines and chemokines to play an important role in inflammatory skin diseases [27, 28]. Our results demonstrated a higher expression level of pro-inflammatory cytokines such as TNFα, IL-8 and CXCL1 in MKCs and in HKCs after the disruption of calcineurin signaling (Fig 2A–2D), which was confirmed by ELISA analysis for the secretion of cytokines in the medium (Fig 2E–2G). Interestingly, keratinocytes showed an increased production of pro-inflammatory cytokines caused by the disruption of calcineurin signaling even without TPA or UVB treatment (Fig 2). These results suggest that calcineurin signaling plays an intrinsic role in negatively regulating cytokine production in keratinocytes.

### Calcineurin down-regulates cytokine production by controlling the stabilization of TTP in keratinocytes

The production of cytokines is regulated at various cellular levels including transcriptional, post-transcriptional and translational levels [29]. Since calcineurin signaling activates NFAT to positively control the transcription of immune response genes, including cytokines, in T cells [6], the negative regulation of cytokine production by calcineurin in keratinocytes likely acts at the post-transcriptional level. Indeed, our results demonstrate that the well-characterized RNA binding protein TTP is significantly down-regulated in MKCs and in HKCs by the inhibition of calcineurin (Fig 3A and 3B). The down-modulation of TTP by calcineurin was observed both in experimental tumors and in clinical SCCs. Importantly, regulation of the TTP expression level by calcineurin in keratinocytes is specific, because other RNA-binding proteins were not affected by the deletion of CnB1. Moreover, we found that the down-modulation of TTP was sufficient to recapitulate the effects of calcineurin signaling inhibition, and the over-expression of TTP decreased the production of those cytokines. This suggests that calcineurin controls cytokine production through TTP in keratinocytes.

The regulation of TTP occurs at multiple levels of cellular signaling events from transcription, mRNA turnover, and phosphorylation status to proteasomal degradation [16]. At the level of transcription, the gene encoding TTP has been reported to be under the control of TGF-β/Smad signaling [30]. We found that the inhibition of calcineurin by knockdown of CnB1 or CsA, but not by blocking calcineurin downstream targets NFATs, caused a decrease of TTP protein levels while it increased rather than decreased TTP mRNA levels, which might be due to the fact that TTP negatively regulates the stability of its own mRNA [31]. This suggests that the inhibition of calcineurin affects the stabilization of TTP rather than transcriptionally regulating its expression in keratinocytes. However, we can’t completely exclude the possible role of NFAT controlling TTP expression since there are four NFAT isoforms that have functional redundancy. We further found that in cells with the CnB1 deletion, the TTP protein is destabilized and shows a reduced half-life. This result was confirmed by the fact that the destabilization of TTP by the inhibition of calcineurin could be blocked by the proteasome inhibitors epoxomicin or MG132 (Fig 5D and 5E), and proteasome inhibitors didn’t alter the mRNA level of TTP in keratinocytes treated with CsA. These data suggest that calcineurin signaling regulates TTP stabilization through a proteasome-mediated degradation mechanism. Current evidence shows that the phosphorylation status of TTP is important for its stability and activity [16]. The TTP protein can be extensively phosphorylated by multiple signaling pathways: ERK/MAPK, p38 MAPK, JNK and AKT. p38 MAP-activated protein kinase 2 (MK-2) *in vitro* and *in vivo*, and protein phosphatase 2 (PP2A) can dephosphorylate TTP [32, 33]. Taken together, we conclude that calcineurin can control the stabilization of TTP through proteasome-mediated degradation, which likely results from a change of its phosphorylation profile. However, the detailed mechanism involved still needs further studies.

### TTP, a downstream target of calcineurin, plays a tumor suppressing function in keratinocytes

The aberrant over-expression of ARE-containing genes plays a crucial role in the initiation and progression of tumorigenesis [22, 34, 35]. A loss or decreased expression of TTP was observed in various epithelial tumors including breast, cervix, colon, liver, prostate and other cancers [18, 22, 23, 35, 36]. More and more evidence shows that the loss of TTP promotes tumorigenesis through multiple cancer-associated progressions including enhancing cancer cell proliferation by regulating IL-8, VEGF and COX2 production, and accelerating the cell cycle [18]. Recently, it was reported that TTP regulates the metabolic process of prostate tumor cells. In agreement with previous reports, our study also demonstrates that TTP is down-regulated in clinical tumor-derived skin and in oral SCC keratinocyte cell lines, as well as in clinical SCCs compared to normal skin. The knockdown of cellular TTP promotes keratinocyte tumorigenesis, possibly through multiple pathways including increased IL-8, VEGF and COX2 production, and the alteration of metabolic processes of skin tumor cells. These findings support the notion that TTP plays a tumor-suppressing role in the skin. Importantly, down-regulated levels of TTP in skin tumors correspond to the inhibition of calcineurin signaling both in experimental tumors and in clinical SCCs. Notably, the overexpression of TTP could partially block tumorigenesis by inhibiting calcineurin signaling. This suggests that the negative regulation of skin inflammation through TTP stabilization could also contribute to the tumor suppressing function of calcineurin. Future studies will focus on exploring the detailed molecular mechanisms of TTP as a tumor suppressor in keratinocytes.

### Conclusions

In summary, we discovered that calcineurin negatively controls pro-inflammatory cytokine production by controlling the stabilization of TTP both in MKCs and in HKCs. The disruption of calcineurin signaling facilitates the degradation of TTP, which results in an enhanced pro-inflammatory cytokine/chemokine production, increased skin inflammation and keratinocyte tumorigenesis. The down-regulation of TTP by calcineurin inhibition in keratinocytes could contribute to increased skin inflammation and SCC development in organ-transplanted patients. As a downstream target, TTP plays an important role in the tumor suppressing function of calcineurin.

## Methods

### Ethics statement

The procedure for obtaining human foreskin tissues from discarded hospital specimens was reviewed and approved by the Medical Ethical Committee of the School of Stomatology, Shandong University (Protocol NO: 2015120401, Date: 12-05-2015). Specimens were analysed anonymously and no patient consent was required.

All animal studies were approved by the ethics committee of Stomatological Hospital Shandong University, (Protocol NO.2015120402, Date: 12-05-2015). All the animal procedures in this study were carried out in accordance with National Institutes of Health Guidelines for the Care and Use of Laboratory Animals.

### Cell culture, virus infection, reagents and human samples

MKCs were prepared from newborn mice and were cultured following standard protocols, as previously described [37, 38]. In order to obtain CnB1 KO MKCs, MKCs from CnB1^flox/flox^ transgenic mice (see below) were infected with an adenovirus expressing Cre (Ad-Cre) or GFP (Ad-GFP, as a control). Primary HKCs were isolated from foreskin tissues and were cultured in serum-free keratinocyte medium (SFM, Invitrogen) as previously described [39–41]. All SCC lines (SCC12, 13, 15, 25 were derived from the skin, and SCCO11, O12, O22, O23, O28 were derived from the oral epithelium) were cultured in SFM. Human foreskin tissues were obtained from discarded hospital specimens following an institutional protocol (NO.2015120401, Date: 12-05-2015). Clinical SCC samples were obtained from the Department of Dermatology, Zurich University Hospital, Zurich, Switzerland. Participants were provided verbal and written informed consent; the protocol was approved by the Swiss Ethics Committee as described previously [26, 42].

Methods for infecting adenoviruses, lentivirus or retroviruses followed described protocols [26, 43]. For transient transfection of siRNA, Lipofectamine 2000 (Invitrogen) was employed and the final concentration of siRNA in the transfection medium was 200 nM. All siRNA oligo sequences are listed in S2 Table.

CsA (30024, Sigma-Aldrich) was dissolved in DMSO and stored as a stock solution (20 mM); TPA (P8139, Sigma-Aldrich) was dissolved in acetone and stored as a stock solution (10 mM). Both CsA and TPA were kept at -80°C. The Vivit peptide, an inhibitor of calcineurin mediated NFAT activation [44], and its negative control, the Veet peptide, were chemically synthesized by the Peptide Core facility of the University of Lausanne and were then dissolved in H_2_O and stored as stock solutions (20 mM) at -80°C. The final concentration of CsA and Vivit used to treat in vitro keratinocyte cultures was 5 μm.

For UVB treatment *In vitro*: After removal of culture medium and two washes with PBS, confluent keratinocytes were covered with PBS and exposed to 35 mJ/cm^2^ UVB. The UVB dose was measured each time using an IL 1400A photometer (International Light Inc., Newburyport, MA) equipped with a SEL240 probe. After UVB exposure, PBS was removed from the cells and replaced with culture medium. Cells were harvested at different times (as indicated in each experiment) after UVB treatment for RNA isolation and total protein extract preparation.

### Animal studies

#### Skin inflammation models

8 week-old female C57BL/6 mice were used for *in vivo* inflammation studies. The CnB1 transgenic mice were derived from a previous study [3]. Wild-type mice with the *CnB1* gene flanked by two lox-p sites were labeled with “CnB1^flox/flox^” as the control group, and mice with *CnB1* gene knockout in keratinocytes (CnB1^flox/flox^+K14cre) were labeled as “CnB1 KO”.

For ear treatment, TPA was dissolved in acetone (100 μg/ml); one ear was treated with a single dose of 20 μl TPA, and the other ear was treated with 20 μl acetone. Both wild-type and KO mice were used in these studies. Ear thickness was measured at different time points using a digital caliper (Mitutoyo Corp., Tokyo, Japan). At the end of the experiment, mice were sacrificed and one-half of each ear was excised and snap-frozen in liquid nitrogen for RNA isolation, and the other half of each ear was embedded in OCT compound for frozen section histology.

For UVB treatment, after mice were anesthetized and shaved, the dorsal skin was treated with Nair cream, then half of the dorsal skin was exposed to UVB at a dose of 160 mJ/cm^2^ while the other half was covered and was not exposed to UVB as a control. Skins were harvested 24 or 48 hr later after the mice were sacrificed and were processed either for immunohistochemical analysis or for epidermal separation and total RNA preparation (ad detailed below).

#### Tumorigenicity assays

Intradermal tumorigenicity studies were performed as previously described [26, 45]. Briefly, HKCs were transfected with siRNAs against TTP or scrambled siRNA controls (100 nM siRNA in HiPerFect transfection reagent, Qiagen) for 12 hr, then were infected with a H-*ras*^V12^ transducing retrovirus (LZRS-ras^V12^) [46]. Sixteen hr after infection, cells were suspended and injected (10^6^ cells per injection, two side flank injections per mouse) into the dermal-epidermal junction of 8 week old female nu/nu mice (Charles River). Six weeks after injection, mice were sacrificed for analysis. To define the lesion according to our previous study [26]. *H-ras*^*V12*^-expressing HKCs produced an acanthotic epithelium with a normal pattern of differentiation, while the lesion was characterized with disordered proliferation, cellular and nuclear pleomorphism, and histopathological and immunohistochemical features similar to moderately to poorly differentiated SCC.

In an alternative approach, HKCs were first infected with lentivirus expressing TTP; 24 hr later they were infected with a H-*ras*^V12^ transducing retrovirus, and 12 hr later, cells were collected and injected into the mice. Starting at 24 hr after the injection of cells, mice were given I.P. injections, every other day, of CsA (20 μg/g body weight in DMSO) or were treated with vehicle alone as a control. Six weeks later, the mice were sacrificed and assessed for tumor formation.

### Quantitative real time RT-PCR, immunodetection techniques and tissue microarray

Procedures for real time RT-PCR analysis, immunoblotting and immunofluorescence (IF) analysis were executed as previously described [47–49]. The list of gene-specific primers is provided in S1 Table. We used the following primary antibodies: polyclonal rabbit anti-TTP (ab33058, for IF staining), monoclonal rat anti-F4/80 (ab16911), monoclonal rat anti-F4/80 (Ly6c) (ab15627), polyclonal rabbit anti-CD4 (ab183685) and rabbit monoclonal anti-Ki67 (ab16667) were purchased from Abcam; polyclonal rabbit anti-CD8 (98941) was purchased from Cell Signalling: polyclonal goat anti-TIA-1 (sc-1751), mouse monoclonal anti-TIAR (sc-398372), mouse monoclonal anti-HuR (sc-5261), mouse monoclonal anti-TNFα (sc52746) and mouse monoclonal anti-γ-tubulin (sc-17787) were purchased from Santa-Cruz; mouse monoclonal anti-CD16 (NBP2-42228) and mouse monoclonal anti-CD161 (NB100-77528) were purchased from Novus Biological. Mouse monoclonal anti-CnB1 (C0581) and anti-ZFP36/TTP (SAB4200565, for Western-blot)) were purchased from Sigma.

Tissue Microarray (TMA) analysis was performed as previously described [26]. Eighteen *in situ* SCC samples from immunocompetent patients, 16 *in situ* SCCs from CsA treated patients and 12 normal skin samples form immunocompetent patients were analyzed for TTP expression by immunohistochemistry. All staining was performed twice and was evaluated twice by two Independent persons. Evaluations were based on arbitrary units as follows: 1—no or weak staining, 2—intermediate staining and 3—strong staining. For the relative quantification of TTP expression, the mean value of the independent measurements was taken as the final score.

### ELISA

Elisa kits to measure secreted cytokines human IL-8 (VAL103), human TNFα (VAL105) and mouse TNFα (MTA00B) were purchased from R&D Systems, Inc (Minneapolis, MN, USA), and the detection procedure followed the manufacturer’s protocol.

### Analysis of protein stability

For protein stability assays, puromycin (30 μg/ml in DMSO) was added to the cell medium to inhibit translation starting 3 hr after UVB treatment. Cells were collected immediately before and 1, 2 and 4 hr after the addition of puromycin. Cells were then lysed in loading buffer for Western blot analysis, and protein half-lives were extrapolated from densitometric analysis of the electrophoretic bands through linear regression.

### Proteasome inhibition assay

To duplicate 60 mm dishes, either 1 μM Epoxomicin (#24801, EMD), 10 μM MG132 or vehicle (DMSO) was added directly to the cell medium, starting 3 hr after UVB treatment and subsequently after an additional 2 and 5 hr. Cells were collected at the indicated times and then lysed in loading buffer for Western blot analysis.

## Supporting information

S1 Fig Increased inflammation in skin tumors elicited by deletion of the *CnB1* gene in keratinocytes. **A.** Multistep skin carcinogenesis (DMBA/TPA) of mice with keratinocyte-specific CnB1 gene deletion (KO) together with littermate controls (Ctrl). Tumor sections were analyzed by immunofluorescence for the infiltration of inflammatory cells using antibodies against Gr-1 (red) and F4/80 (red). DAPI (blue) was used as a nuclear counter-stain. **B.** Quantification of Gr-1 or F4/80 positive cells in the tumor tissues shown in **A**. ** p<0.01, n = 6.
(JPG)

S2 Fig No difference of T cell populations present in skin tumors with deletion of the *CnB1* gene in keratinocytes compared to control mice. **A-D.** The tumor sections (both the Ctrl and the KO group) shown in S1 Fig were analyzed by immunofluorescence for the infiltration of different T cell populations using antibodies against CD4 (red) in **A**, CD8 (red) in **B**, CD16 (red) in **C** and CD161 (red) in **D**. DAPI (blue) was used as a nuclear counter-stain. Quantification of positive cells infiltrating the tumor tissues is shown in the panels on the right. Six mice (n = 6) were analyzed from each group.
(TIF)

S3 Fig Increased inflammation in skin tumors with disruption of CnB1 signaling in keratinocytes either by knockdown of CnB1 or by CsA. **A.** Tumors were generated by injecting immunocompromised mice with H-*ras*^V12^ expressing HKCs treated with inhibitors of calcineurin, i.e. a siRNA of CnB1 (siCnB1) or with CsA treatment, or with a scramble siRNA (siCtrl). Tumor sections were analyzed by immunofluorescence for the infiltration of inflammatory cells using an antibody against Gr-1 (red), with DAPI (blue) as a nuclear counter-stain. **D.** Quantification of Gr-1 positive cells in the tumor tissues shown in **B**. ** p<0.01, n = 6.
(TIF)

S4 Fig No adaptive immune response is triggered in skin treated with TPA for 48 hr. **A-D**: Skin sections from Ctrl and KO mice treated with TPA for 48 hr were analyzed by immunofluorescence for the infiltration of different T cell populations using antibodies against CD4 (red) in **A**, CD8 (red) in **B**, CD16 (red) in **C** and CD161 (red) in **D**. DAPI (blue) was used as a nuclear counter-stain.
(TIF)

S5 Fig The deletion of *CnB1* down-regulates TTP protein levels in keratinocytes. **A:** Quantification of band densities in immunoblots of RNA-binding proteins TTP, TIA-1, TIAR and HuR shown in Fig 3A. ***p<0.001, Student’s t test, n = 3. **B:** Quantification of IF staining of TTP shown in Fig 3B. The evaluation of TTP staining was done based on arbitrary units with the following scores: 0-negative staining, 1-weak staining, 2-intermediate staining and 4-strong staining. The mean score of TTP staining was calculated by evaluating 100 cells based on DAPI nuclear staining for each group; the experiment was repeated 3 times (n = 3), **p<0.01.
(TIF)

S6 Fig Decreased expression of TTP is found in SCC cell lines. The full-blot image of the immunoblot shown in Fig 3E. Two different primary human keratinocyte lines (HKC1 and HKC2) plus 10 SCC cell lines were analyzed for TTP expression by immunoblotting; tubulin served as a loading control. The markers indicate the bands of TTP and tubulin proteins.
(TIF)

S7 Fig TTP protein level is significantly knocked down by siRNA. The full-blot image of the immunoblot shown in Fig 4B. Three different siRNAs of TTP (siTTP-1,2,3) and two scrambled siRNA (siCtrl1,2) were transfected into HKCs. Seventy-two hr after transfection, the cells were collected for TTP analysis by immunoblotting; tubulin was used as a loading control. The molecular weights of TTP and tubulin are indicated.
(TIF)

S8 Fig The reduced half-life of TTP protein in keratinocytes with deletion of CnB1. The half-life of the TTP protein was extrapolated from the densitometric analysis of the electrophoretic bands shown in Fig 5E through linear regression.
(TIF)

S9 Fig TTP affects the expression of cytokines and genes involved in metabolic pathways in SCC13 skin tumor cells. **A-D**: qRT-PCR analysis of mRNA levels of indicated cytokines and metabolic genes in SCC13 skin tumor cells either with knockdown of TTP (siTTP) in **A, B** or with over-expression of TTP (TTP-OE) in **C, D**. PCR results were normalized with the 36B4 gene. Error bars indicate standard error, Student’s t test, n = 3, *p<0.05, **p<0.01 when compared to the corresponding controls (white bars).
(TIF)

S1 Table Oligo sequences for real time RT-PCR analysis. (TIF)

S2 Table siRNA sequences for knockdown experiments. (TIF)
