# Supplementary figures and images for: The ARE-binding protein Tristetraprolin (TTP) is a novel target and mediator of calcineurin tumor suppressing function in the skin
Source: PLoS Genet. 2018 May 3;14(5):e1007366. doi: 10.1371/journal.pgen.1007366 (PMC5953486; doi:10.1371/journal.pgen.1007366)

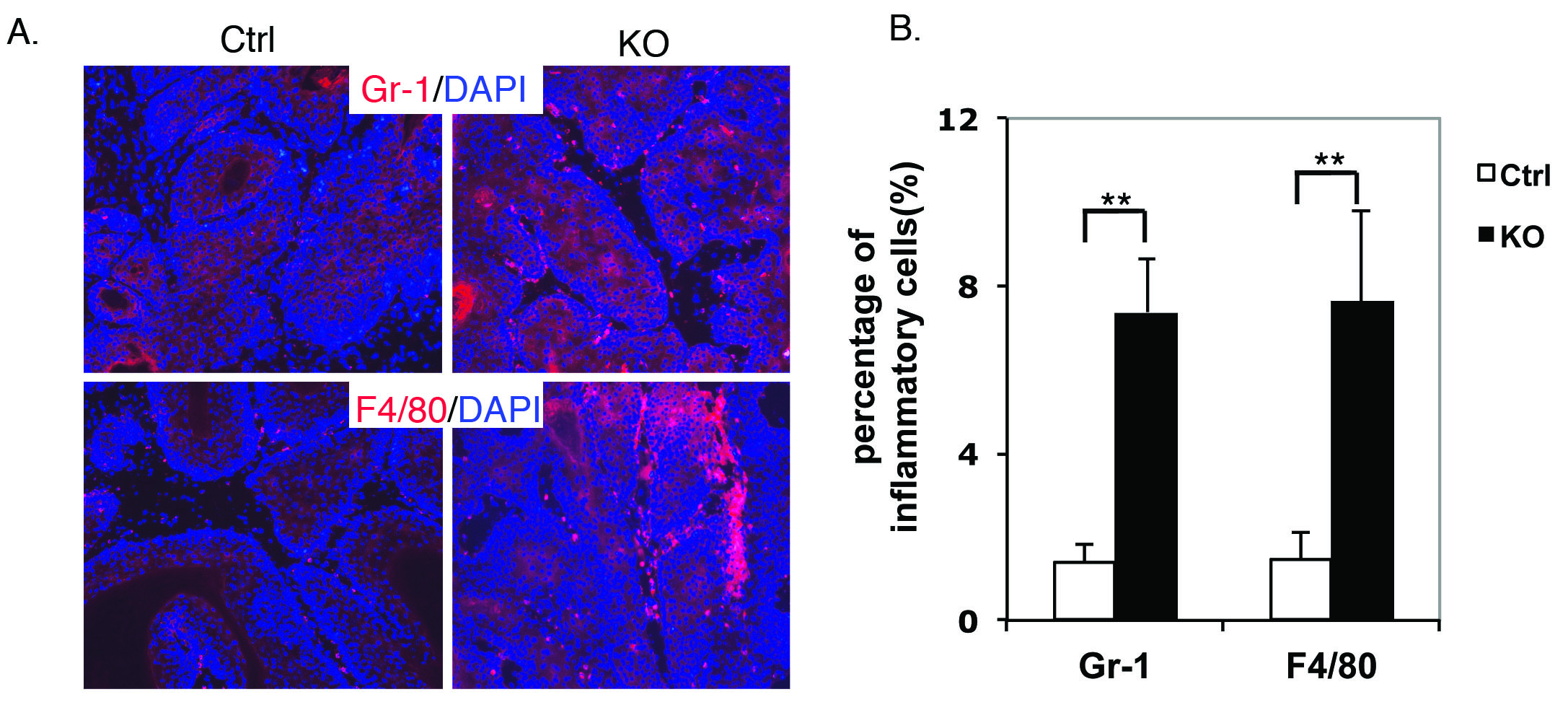

Supplement: S1 Fig — A. Multistep skin carcinogenesis (DMBA/TPA) of mice with keratinocyte-specific CnB1 gene deletion (KO) together with littermate controls (Ctrl). Tumor sections were analyzed by immunofluorescence for the infiltration of inflammatory cells using antibodies against Gr-1 (red) and F4/80 (red). DAPI (blue) was used as a nuclear counter-stain. B. Quantification of Gr-1 or F4/80 positive cells in the tumor tissues shown in A. ** p<0.01, n = 6. (JPG) [file pgen.1007366.s001.jpg]

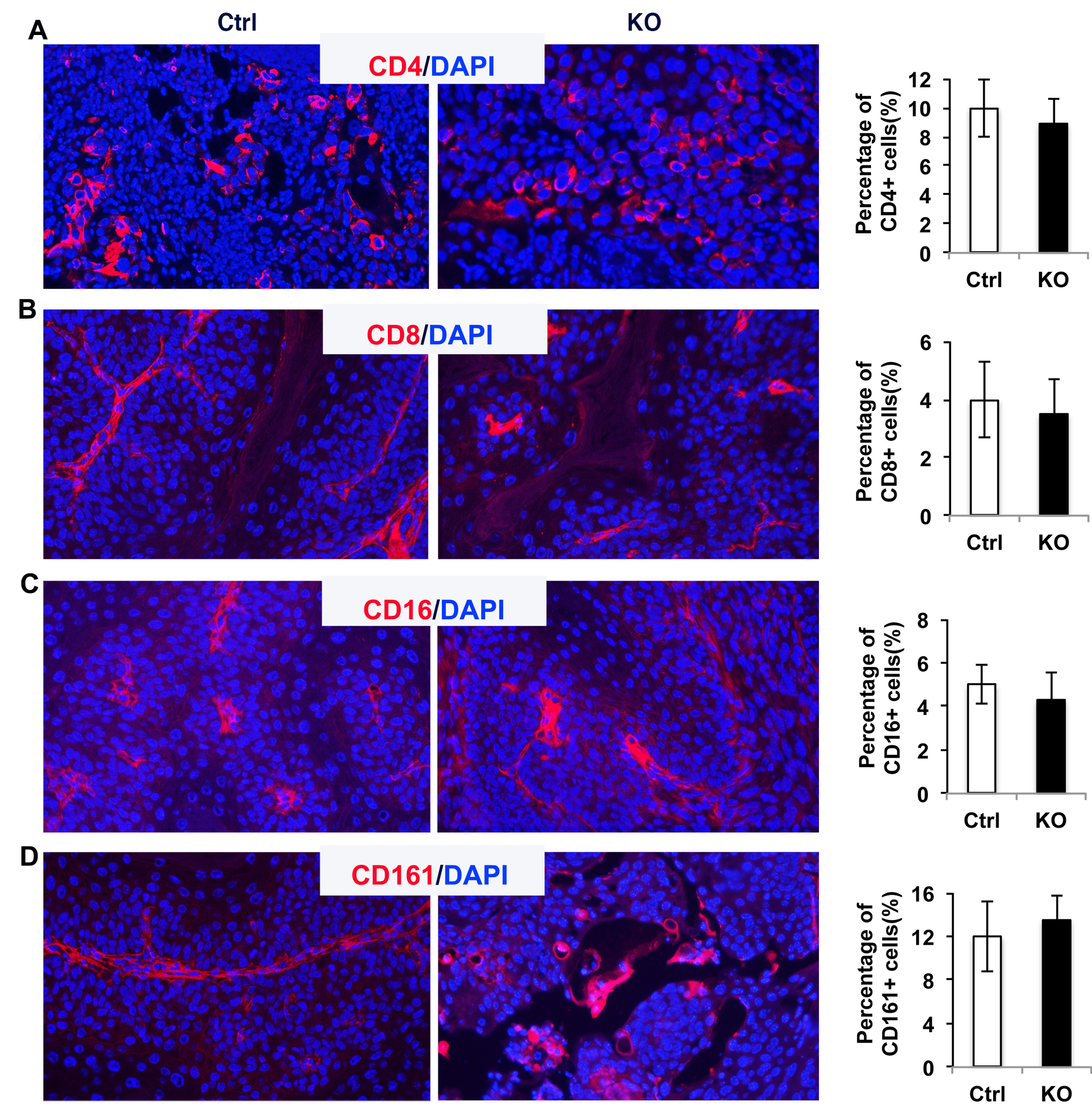

Supplement: S2 Fig — A-D. The tumor sections (both the Ctrl and the KO group) shown in S1 Fig were analyzed by immunofluorescence for the infiltration of different T cell populations using antibodies against CD4 (red) in A, CD8 (red) in B, CD16 (red) in C and CD161 (red) in D. DAPI (blue) was used as a nuclear counter-stain. Quantification of positive cells infiltrating the tumor tissues is shown in the panels on the right. Six mice (n = 6) were analyzed from each group. (TIF) [file pgen.1007366.s002.tif]

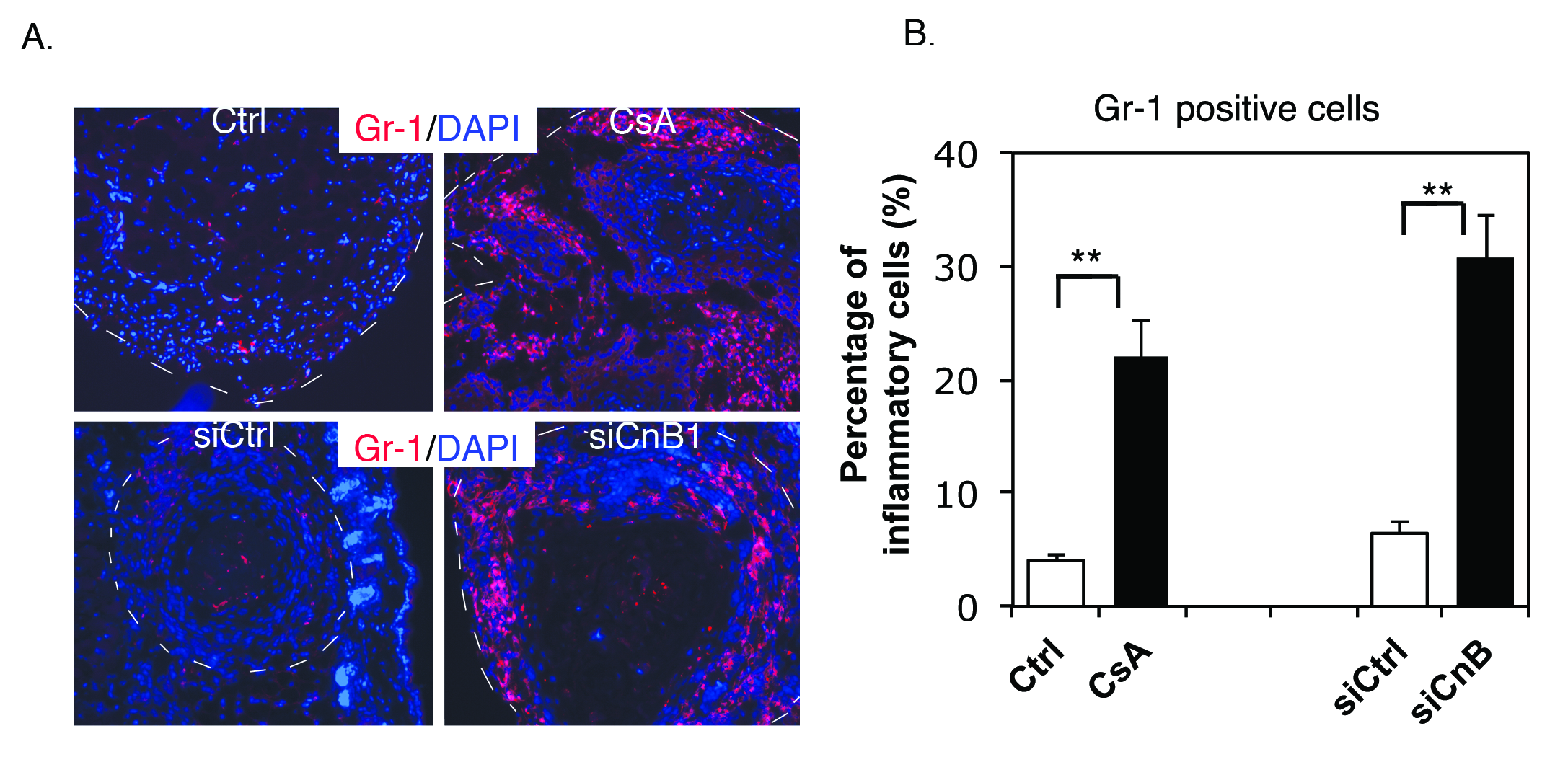

Supplement: S3 Fig — A. Tumors were generated by injecting immunocompromised mice with H-rasV12 expressing HKCs treated with inhibitors of calcineurin, i.e. a siRNA of CnB1 (siCnB1) or with CsA treatment, or with a scramble siRNA (siCtrl). Tumor sections were analyzed by immunofluorescence for the infiltration of inflammatory cells using an antibody against Gr-1 (red), with DAPI (blue) as a nuclear counter-stain. D. Quantification of Gr-1 positive cells in the tumor tissues shown in B. ** p<0.01, n = 6. (TIF) [file pgen.1007366.s003.tif]

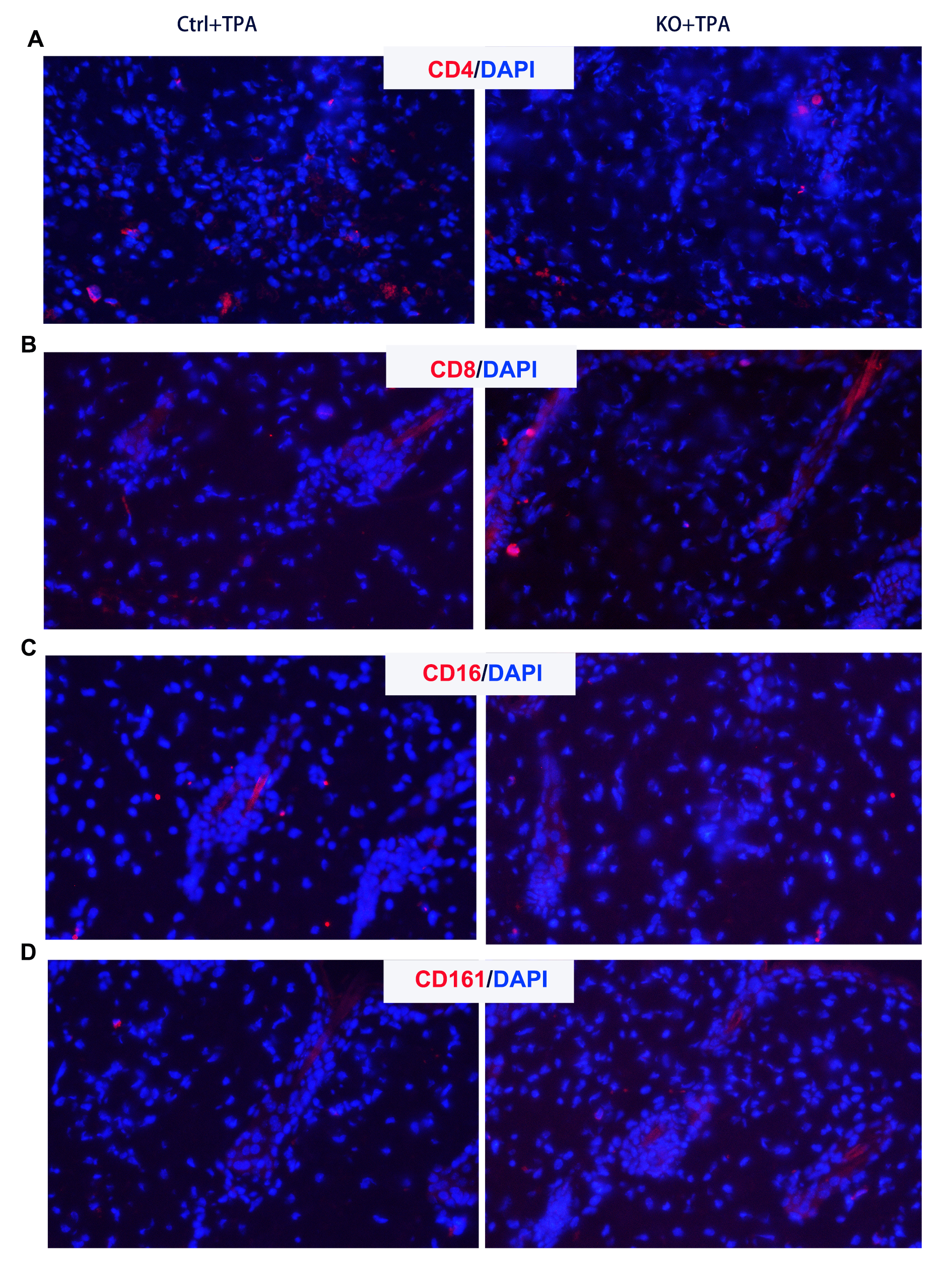

Supplement: S4 Fig — A-D: Skin sections from Ctrl and KO mice treated with TPA for 48 hr were analyzed by immunofluorescence for the infiltration of different T cell populations using antibodies against CD4 (red) in A, CD8 (red) in B, CD16 (red) in C and CD161 (red) in D. DAPI (blue) was used as a nuclear counter-stain. (TIF) [file pgen.1007366.s004.tif]

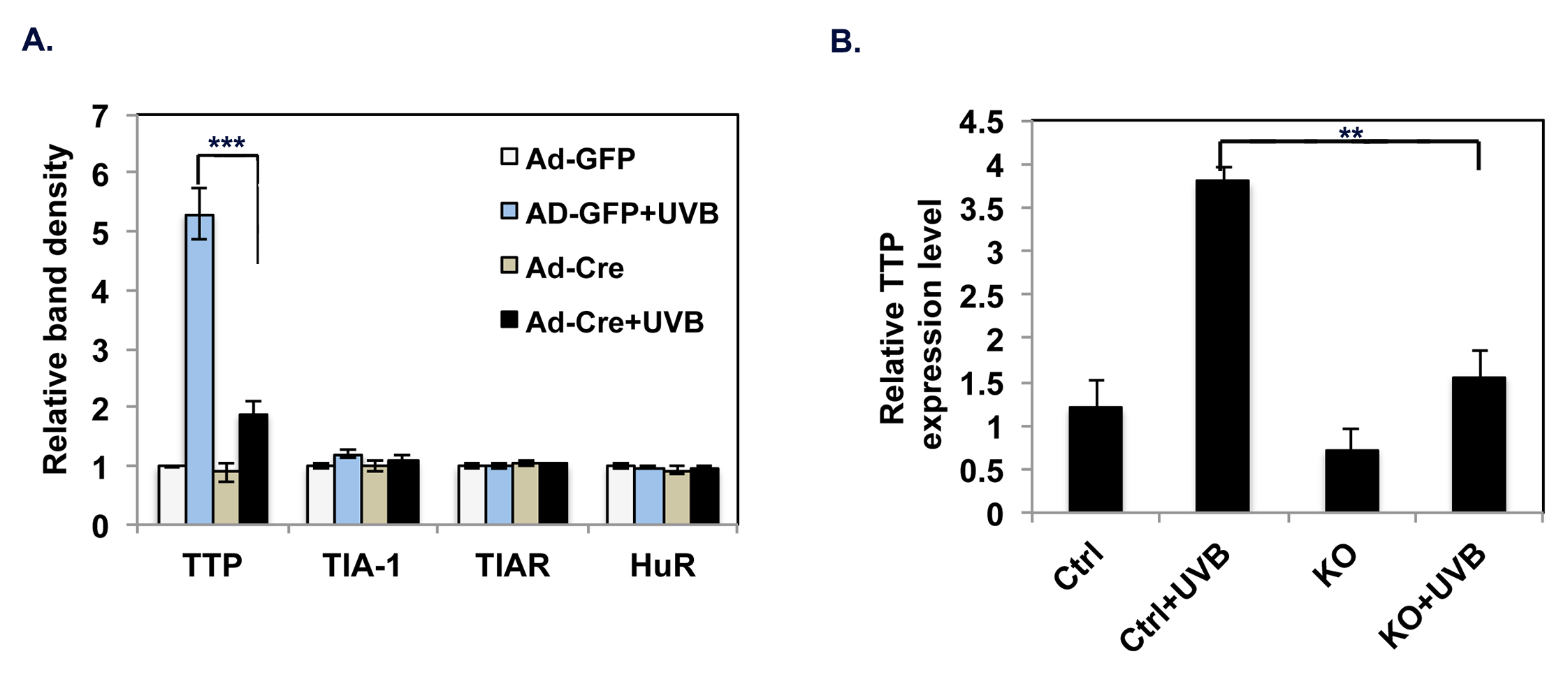

Supplement: S5 Fig — A: Quantification of band densities in immunoblots of RNA-binding proteins TTP, TIA-1, TIAR and HuR shown in Fig 3A. ***p<0.001, Student’s t test, n = 3. B: Quantification of IF staining of TTP shown in Fig 3B. The evaluation of TTP staining was done based on arbitrary units with the following scores: 0-negative staining, 1-weak staining, 2-intermediate staining and 4-strong staining. The mean score of TTP staining was calculated by evaluating 100 cells based on DAPI nuclear staining for each group; the experiment was repeated 3 times (n = 3), **p<0.01. (TIF) [file pgen.1007366.s005.tif]

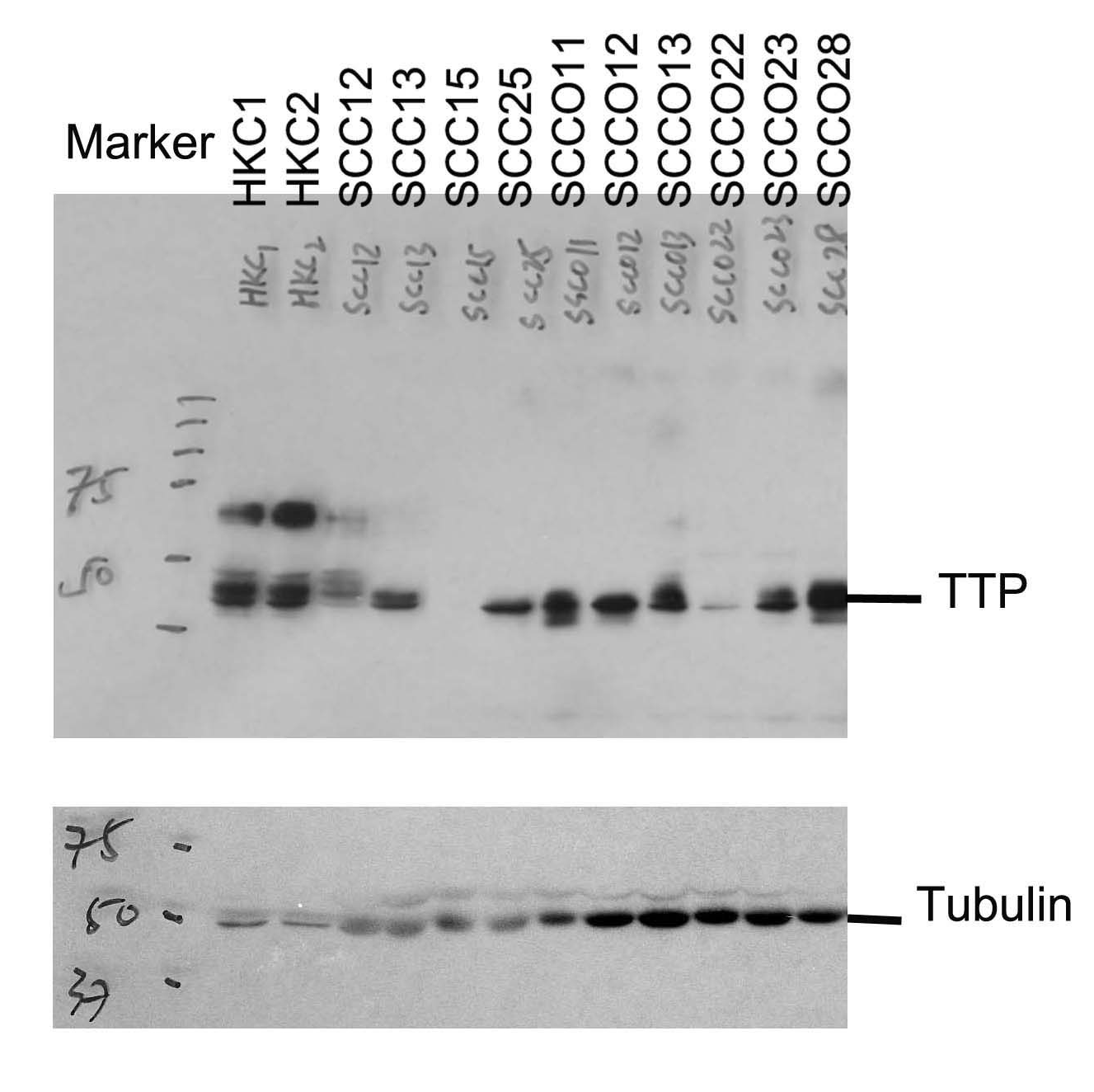

Supplement: S6 Fig — The full-blot image of the immunoblot shown in Fig 3E. Two different primary human keratinocyte lines (HKC1 and HKC2) plus 10 SCC cell lines were analyzed for TTP expression by immunoblotting; tubulin served as a loading control. The markers indicate the bands of TTP and tubulin proteins. (TIF) [file pgen.1007366.s006.tif]

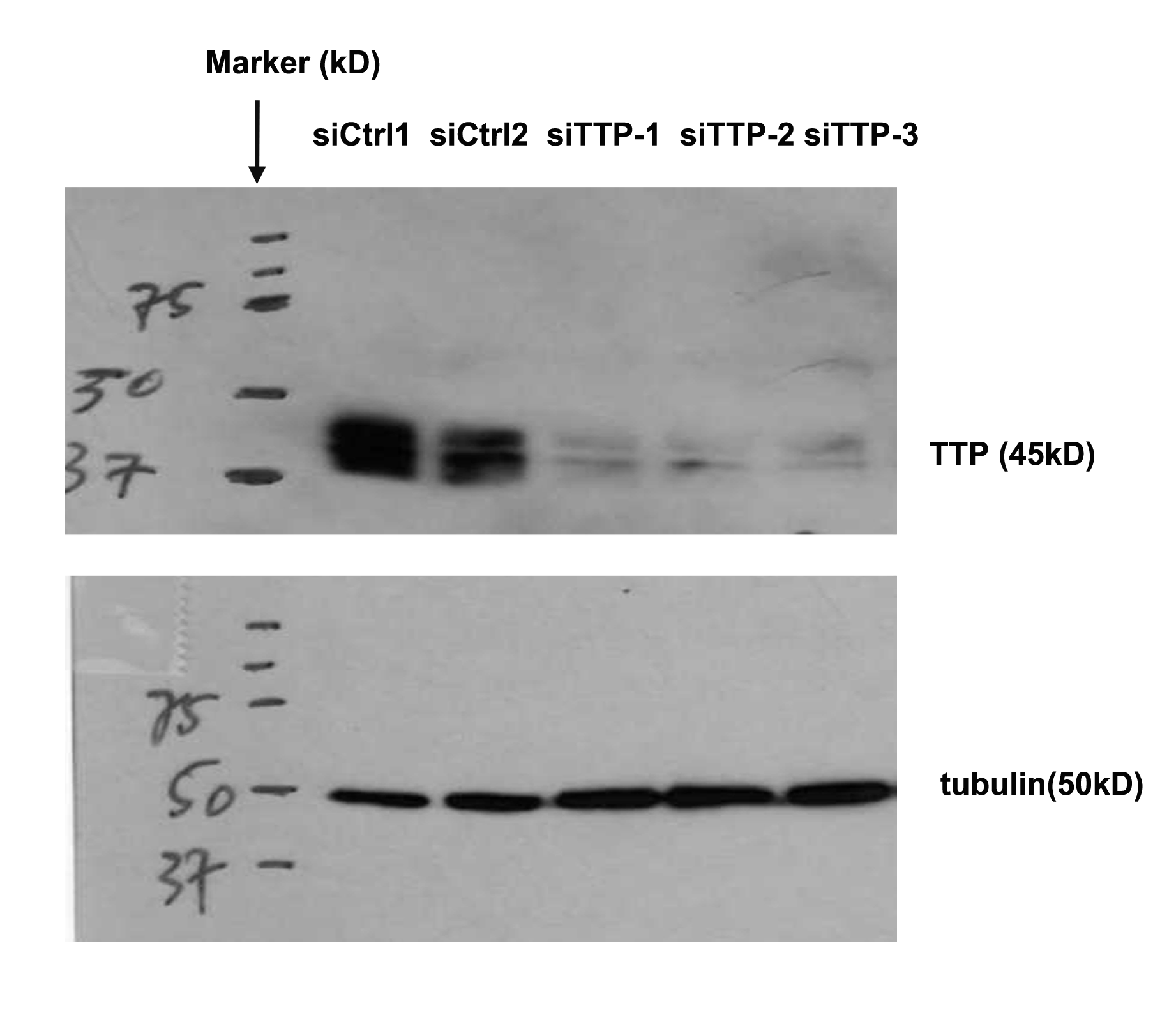

Supplement: S7 Fig — The full-blot image of the immunoblot shown in Fig 4B. Three different siRNAs of TTP (siTTP-1,2,3) and two scrambled siRNA (siCtrl1,2) were transfected into HKCs. Seventy-two hr after transfection, the cells were collected for TTP analysis by immunoblotting; tubulin was used as a loading control. The molecular weights of TTP and tubulin are indicated. (TIF) [file pgen.1007366.s007.tif]

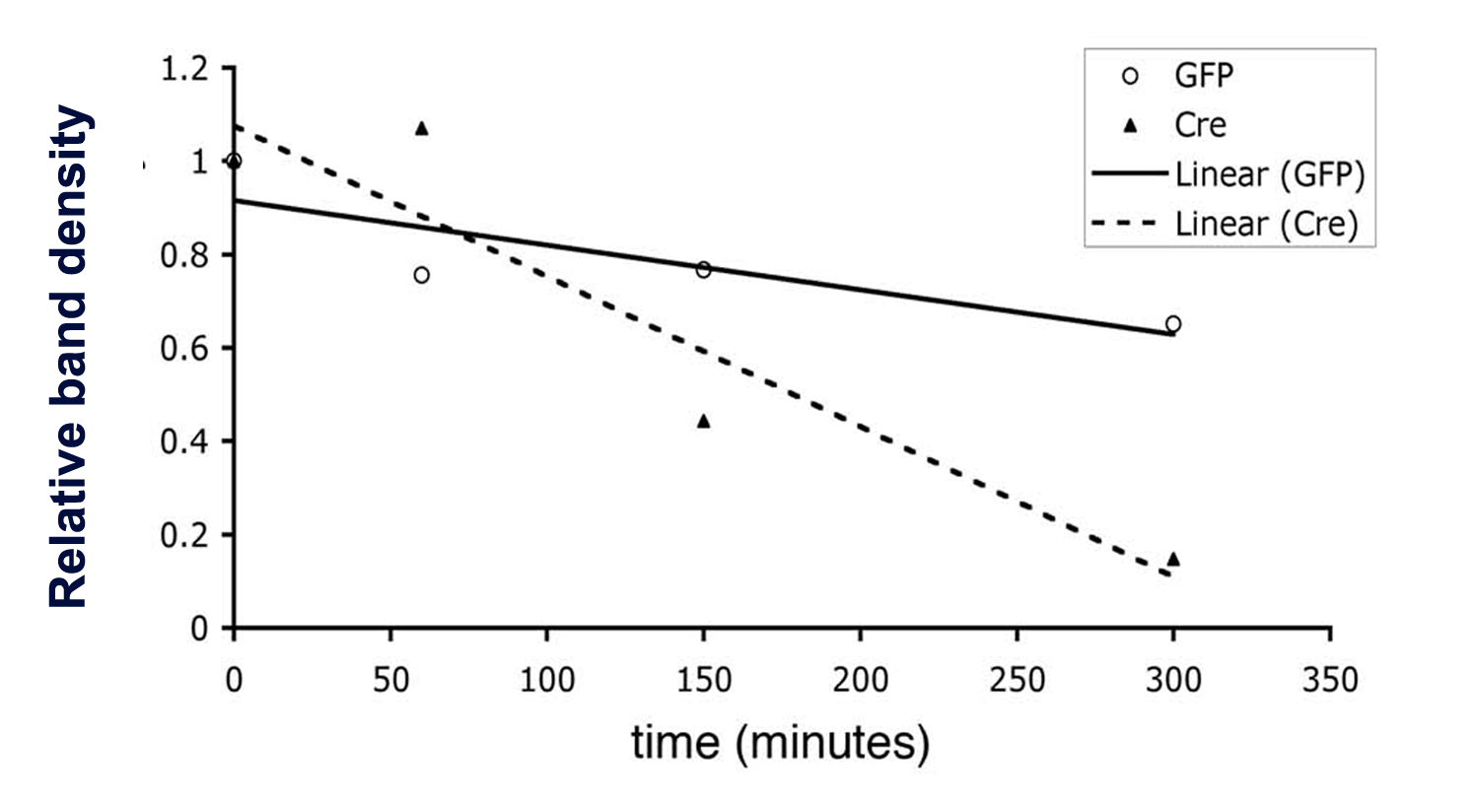

Supplement: S8 Fig — The half-life of the TTP protein was extrapolated from the densitometric analysis of the electrophoretic bands shown in Fig 5E through linear regression. (TIF) [file pgen.1007366.s008.tif]

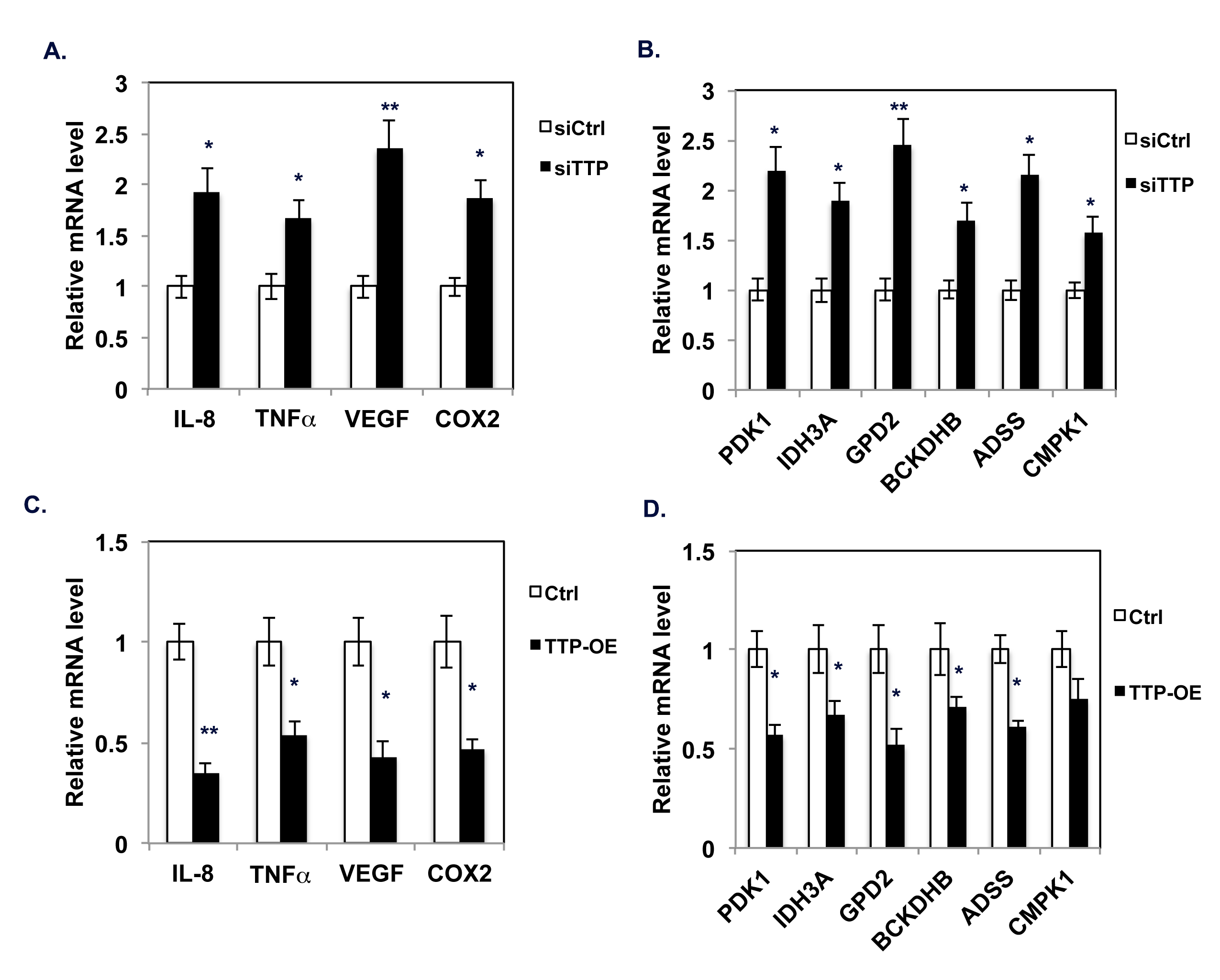

Supplement: S9 Fig — A-D: qRT-PCR analysis of mRNA levels of indicated cytokines and metabolic genes in SCC13 skin tumor cells either with knockdown of TTP (siTTP) in A, B or with over-expression of TTP (TTP-OE) in C, D. PCR results were normalized with the 36B4 gene. Error bars indicate standard error, Student’s t test, n = 3, *p<0.05, **p<0.01 when compared to the corresponding controls (white bars). (TIF) [file pgen.1007366.s009.tif]

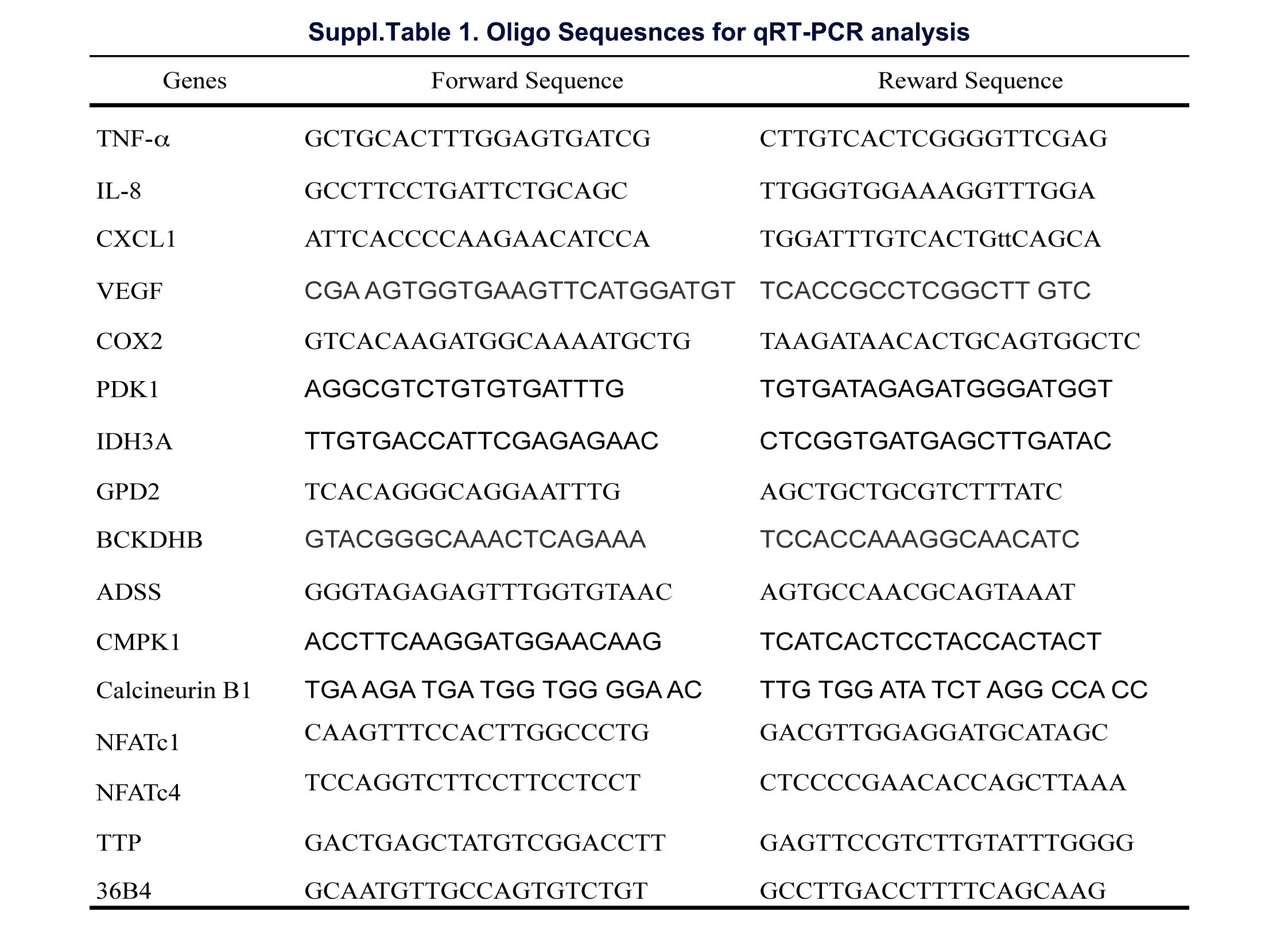

Supplement: S1 Table — (TIF) [file pgen.1007366.s010.tif]

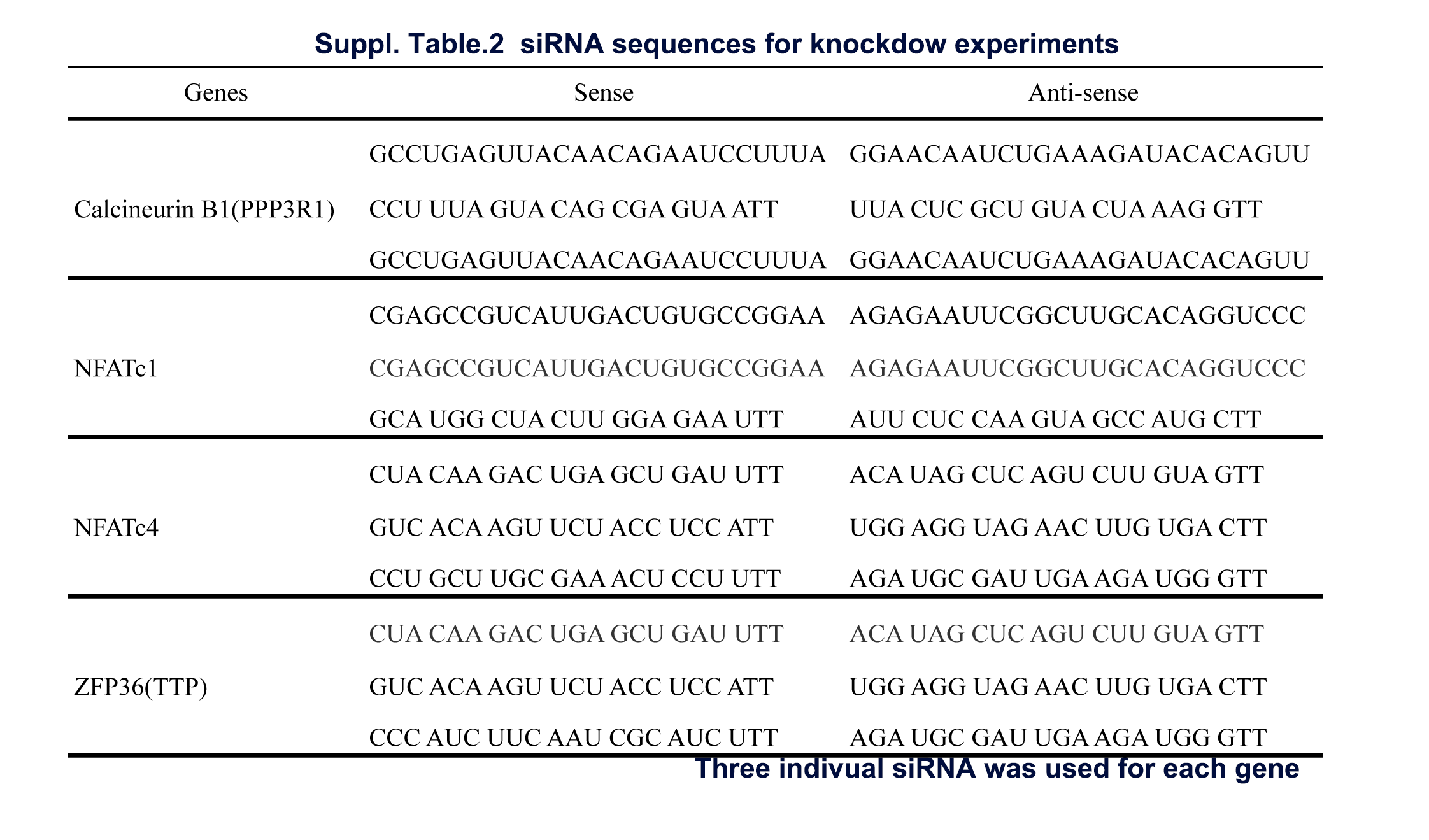

Supplement: S2 Table — (TIF) [file pgen.1007366.s011.tif]
